# Metabolic Impact of Anticancer Drugs Pd_2_Spermine and Cisplatin on the Brain of Healthy Mice

**DOI:** 10.3390/pharmaceutics14020259

**Published:** 2022-01-22

**Authors:** Tatiana J. Carneiro, Martin Vojtek, Salomé Gonçalves-Monteiro, João R. Neves, Ana L. M. Batista de Carvalho, Maria Paula M. Marques, Carmen Diniz, Ana M. Gil

**Affiliations:** 1 Department of Chemistry and CICECO-Aveiro Institute of Materials, University of Aveiro, 3810-193 Aveiro, Portugal; tatiana.joao@ua.pt (T.J.C.); joaodrneves@ua.pt (J.R.N.); 2 LAQV/REQUIMTE, Laboratory of Pharmacology, Department of Drug Sciences, Faculty of Pharmacy, University of Porto, 4150-755 Porto, Portugal; matovoj@gmail.com (M.V.); salomemonteiro8180@gmail.com (S.G.-M.); cdiniz@ff.up.pt (C.D.); 3 Molecular Physical-Chemistry R&D Unit, Department of Chemistry, University of Coimbra, 3004-535 Coimbra, Portugal; almbc@uc.pt (A.L.M.B.d.C.); pmc@ci.uc.pt (M.P.M.M.); 4 Department of Life Sciences, Faculty of Science and Technology, University of Coimbra, 3000-456 Coimbra, Portugal

**Keywords:** palladium(II), platinum(II), spermine, Pd_2_Spm, cisplatin, toxicity, mice, brain extracts, NMR, metabolomics

## Abstract

The new palladium agent Pd_2_Spermine (Spm) has been reported to exhibit promising cytotoxic properties, while potentially circumventing the known disadvantages associated to cisplatin therapeutics, namely acquired resistance and high toxicity. This work presents a nuclear magnetic resonance (NMR) metabolomics study of brain extracts obtained from healthy mice, to assess the metabolic impacts of the new Pd_2_Spm complex in comparison to that of cisplatin. The proton NMR spectra of both polar and nonpolar brain extracts were analyzed by multivariate and univariate statistics, unveiling several metabolite variations during the time course of exposition to each drug (1–48 h). The distinct time-course dependence of such changes revealed useful information on the drug-induced dynamics of metabolic disturbances and recovery periods, namely regarding amino acids, nucleotides, fatty acids, and membrane precursors and phospholipids. Putative biochemical explanations were proposed, based on existing pharmacokinetics data and previously reported metabolic responses elicited by the same metal complexes in the liver of the same animals. Generally, results suggest a more effective response of brain metabolism towards the possible detrimental effects of Pd_2_Spm, with more rapid recovery back to metabolites’ control levels and, thus, indicating that the palladium drug may exert a more beneficial role than cDDP in relation to brain toxicity.

## 1. Introduction

**Figure 1:**
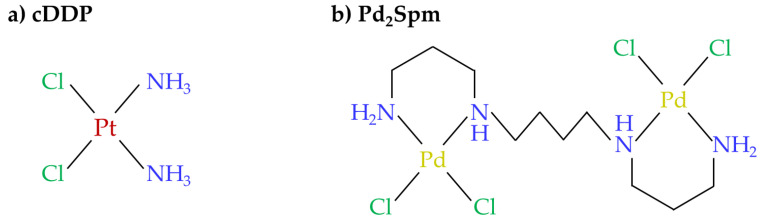
Chemical structures of the (**a**) conventional Pt(II)-drug cisplatin and (**b**) novel complex Pd(II)-spermine (Pd_2_Spm).

Platinum (Pt(II))-based drugs have been used as chemotherapeutic agents in the treatment of several types of solid tumors ever since the discovery of the important antiproliferative properties of cisplatin (cDDP) (Figure 1a) in the mid-1960s [1,2]. However, the treatment regimen of cumulative and high dosage Pt(II)-based drugs is limited by their severe deleterious effects, mostly nephrotoxicity, hepatoxicity, and neurotoxicity [3,4]. Indeed, cDDP is one of the most neurotoxic Pt(II) drugs [5], due to its capacity to progressively form nuclear and mitochondrial adducts with DNA’s purine bases, prompting mitochondrial dysfunction, oxidative stress, and apoptosis of neuronal cells, therefore leading to both central (rare) and peripheral neuropathies [6,7]. The latter have a relatively higher prevalence, affecting about 50% of patients administered with cumulative doses above 300 to 350 mg/m^2^ [3,8]. The peripheral nervous system is damaged by the accumulation of cDDP [6,9], mostly in the dorsal root ganglion neurons, due to (i) the lack of the blood–brain barrier (BBB) and its selective and regulatory role [9], and (ii) overexpression of membrane receptors responsible for cDDP cellular uptake (copper transporter-1 and organic cation transporter-2) [6]. On the other hand, the central nervous system is protected by the BBB, as this is preferentially permeable to small (<15 kDa) and lipophilic molecules and, thus, cDDP is expected to exhibit poor capability to cross this barrier [9,10]. Recently, a study of mice exposure to a single cDDP injection (3.5 mg/kg) supported this information, revealing a significantly lower in vivo biodistribution of Pt(II) in brain tissue (<1 ng/g), compared to those in tissues such as kidney, liver, and lungs (10 to 100 ng/g) [11]. However, other studies have shown that cDDP reaches mice brain upon administration of cumulative doses (2.3 to 10 mg/kg/day, over 10 to 35 days), affecting biological functions of cerebellum and hypothalamus [12,13,14]. Usually, dose adjustment or drug withdrawal are the chosen strategies to minimize the neurotoxic side effects of cDDP [5] and an important research focus has been the identification of potential chemo-protective agents and their mechanisms of action at a molecular level (e.g., curcumin (antioxidant) [15,16], agomelatine (anti-inflammatory) [17], ginkgo biloba (anti-apoptotic effect), metformin (axonal regeneration) [18]).

Palladium [Pd(II)] and gold(I)/(III) complexes are favorable alternatives to Pt(II) drugs, potentially circumventing toxicity and acquired resistance issues associated with Pt(II) agents, while exhibiting promising cytotoxic properties against several types of cancer [19,20]. For instance, Pd(II) chelates with biogenic amines, such as spermine (Spm, H_2_N(CH_2_)_3_NH(CH_2_)_4_NH(CH_2_)_3_NH_2_) (Pd_2_Spm) (Figure 1b), have exhibited encouraging antiproliferative [21,22] and antimetastatic [23] properties, as well as related lower acquired resistance [24] in several tumor cells, namely of breast cancer [21,22,23,25,26], leukemia [25], osteosarcoma [27], squamous [28], and ovarian carcinomas [24]. However, to our knowledge, Pd_2_Spm’s toxic effects on healthy biological systems have been described to a limited extent, although including important biodistribution studies in mice organs [11] and corresponding impact on metabolic profiles [29]. These studies showed that kidney and liver are the most affected organs, with higher Pd(II) accumulation [11] and, consistently, more marked metabolic deviations [29], followed by lungs, ovaries, adipose tissue, and mammary glands [11], the latter exhibiting minor disturbances in metabolism [29]. Further knowledge on the metabolic fate of this and other Pd(II) drugs in biological systems, compared to conventional Pt(II) drugs, may also benefit from recent advances in metallomic strategies [30].

The study of brain-deviant metabolism and its connection to drug-induced neurotoxicity may unveil useful information on the drugs’ mechanisms of action. Most metabolic studies, either in vitro and in vivo, have addressed the evaluation of oxidative stress in the brain, as induced by anticancer agents such as cyclophosphamide [31], cabazitaxel [32], doxorubicin (DOX) [33], methotrexate (MTX) [34], temozolomide (TMZ) [34], oxaliplatin (OXA) [35], and vincristine [36], also including cDDP (assessed both in vivo [15,37,38,39,40] and in vitro [40]). A broadly similar pattern seems to be observed regarding enhanced oxidative stress, expressed by increased levels of reactive oxygen species (ROS) [33,36,37,38] and consequent lipid peroxidation [15,32,33,36,38,39], in tandem with decreased levels of reduced glutathione (GSH) [15,32,38] and deviations in the expression of antioxidant enzymes, namely GSH transferase [15], GSH peroxidase [15], superoxide dismutase [32], and catalase [39]. In this context, metabolomics has emerged as an untargeted analytical approach to characterize the metabolic effect of chemotherapeutic drugs [41], understand possible mechanisms of drug resistance [42], and, ultimately, customize chemotherapy regimens to individual patients through precision personalized medicine strategies [43,44]. Regarding the metabolic profiling of brain tumors, in vitro [45,46,47] and in vivo [48] studies have been conducted to assess treatment response to TMZ [45,48] and cDDP [46,47]. Moreover, cDDP-induced neurotoxicity in healthy Sprague-Dawley rats has also been studied through LC-MS/MS analysis of brain and liver extracts [49], unveiling potential biomarkers, however, requiring an objective compound assignment to attain added information on metabolic pathways. The same technique (in tandem with ^1^H NMR of plasma) was used to analyze the hypothalamus of cachectic Lister-hooded rats to assess cDDP-induced neurotoxicity and evaluate the neuroprotective effect of cannabigerol [50]. Animals exposed only to cDDP revealed dysregulation of the levels of *N*-acyl-γ-aminobutyric acids and lipoamines, mainly *N*-acyl-ethanolamines, which is suggestive of an inhibitory role of this drug on the biosynthetic enzymes of lipoamines. Hence, further metabolomic studies, including NMR-based works, should clearly contribute to a thorough characterization of the brain’s metabolic response to drug exposure, as well as to an accurate assessment of drugs’ performance (anti-tumor effect vs. toxicity). Metabolomic studies have also been conducted for Pd_2_Spm, mainly to evaluate markers of cytotoxicity against (i) MG-63 osteosarcoma cells [51,52] and (ii) triple-negative breast cancer in a xenograft mouse model [53], compared to cDDP [51,52,53] or OXA [52]. Metabolic markers of Pd_2_Spm toxicity have also been sought on kidney, liver, and breast tissues of healthy mice, as previously mentioned, [29] showing a faster metabolic response and recuperation, compared to cDDP, except for the lipophilic metabolism of kidney, which exhibits a delayed response compared to the other tissues. Indeed, Pd_2_Spm-induced variations initially seen in the levels of polar and nonpolar metabolites tend to recover to controls levels between 12 and 48 h, suggesting a reduced negative effect. Moreover, Pd_2_Spm also exhibited favorable pharmacokinetics and beneficial biodistribution in the same animals [11], highlighting the promising profile of this complex. However, the potential neurotoxic effect of Pd_2_Spm needs to be studied and compared to that of cDDP and controls, and this is the aim of the present work.

In this study, potential metabolic markers of brain toxicity of healthy mice exposed to either cDDP or Pd_2_Spm will be evaluated using ^1^H NMR metabolomics of polar and nonpolar extracts of mice brain tissue. To the best of the authors’ knowledge, this is the first of this type of study. The metabolic effects of both complexes are presented at three post-injection times (1, 12, and 48 h) to establish a metabolic time-course response/recovery of mice brain tissue. A correlation with previous reported metabolic variations of liver from the same animals [29] will be advanced to putatively consider the metabolic interaction of the brain–liver axis.

## 2. Materials and Methods

### 2.1. Chemicals and Solutions

Regarding the reagents used: cisplatin (*cis*-dichlorodiammine platinum (II), 99.9%), potassium tetrachloropalladate (II) (K_2_PdCl_4_, 98%), and the amine spermine (*N,N′*-bis(3-aminopropyl)-1,4-diaminobutane, 99%) were obtained from Sigma-Aldrich (Sintra, Portugal). All reagents were of analytical grade. Euthasol^®^ solution (400 mg/mL pentobarbital sodium) was purchased from Le Vet (Oudewater, The Netherlands). The Pd_2_Spm complex was synthesized as described elsewhere [54,55].

### 2.2. Ethical Considerations

All animal handling and care protocols complied with the Portuguese (Decreto-Lei no. 113/2013) and European (Directive 2010/63/EU) legislation for the protection of animals used for scientific purposes, and with the Guide for Care and Use of Laboratory Animals of the National Institutes of Health (NIH). Approval was obtained from the Ethics Committee for Animal Experimentation of the Faculty of Pharmacy of the University of Porto, Porto, Portugal (Permit Number: 25-10-2015), and the Ethics Committee and the Organ Responsible for the Welfare of Animals of ICBAS-UP, Porto, Portugal (Permit number 134/2015). The study also followed the Animal Research: Reporting of In Vivo Experiments (ARRIVE) guidelines [56].

### 2.3. Animals Procedures

Six-weeks-old female BALB/cByJ mice (*n* = 45), Specific-Pathogen-Free (SPF), were obtained from Charles River Laboratories (L’Arbresle, France). The animals were acclimatized for 1 week at the ICBAS-UP Rodent Animal House Facility (Porto, Portugal) and randomly placed in individual ventilated cages (5 animals per cage), containing enrichment material. The mice were housed in the conditions described elsewhere [29]. The animals were randomly divided into three groups (15 animals/group), to be treated via intraperitoneal injection in single doses (200 µL) of either (i) cDDP (3.5 mg/kg body weight, in phosphate-buffered saline solution (PBS)), (ii) Pd_2_Spm (3.0 mg/kg body weight, in PBS and in 1% dimethylsulfoxide (DMSO)) or (iii) vehicle solution (PBS: H_2_PO_4_ 1.5 mM, Na_2_HPO_4_ 4.3 mM, KCl 2.7 mM, NaCl 150 mM, pH 7.4). All solutions injected were sterile filtered. The physical condition of the animals was monitored, and all animals were weighed at the start and end of experiments (20.1 ± 1.7 g and 20.3 ± 1.6 g, respectively). Five animals per group were sacrificed at 1, 12, and 48 h post-injection, with pentobarbital intraperitoneal injection (120 mg/kg) followed by cardiac puncture. One control mouse developed inflammation and was thus excluded from the cohort. Mice brains were excised, snap frozen in liquid nitrogen, and stored (−80 °C) until extraction.

### 2.4. Preparation of Brain Extracts

The frontal cortex of each mouse brain was weighed (0.068 ± 0.0087 g, 0.061 ± 0.013 g and 0.071 ± 0.012 g for controls, cDDP and Pd_2_Spm groups, respectively) and ground using a pestle and mortar, in liquid N_2_ [57,58,59]. Samples were extracted according to the biphasic methanol/chloroform/water (2.0:2.0:1.0) method described elsewhere [60]. The resulting polar and nonpolar phases were vacuum/N_2_ dried separately and stored (−80 °C). Before NMR analysis, aqueous extracts were suspended in 650 µL of 100 mM sodium phosphate buffer (pH 7.4, D_2_O with 0.25% 3-(trimethylsilyl)-propionic-2,2,3,3-d4 acid (TSP)), and lipophilic extracts were suspended in 650 µL of CDCl_3_, with 0.03% tetramethylsilane (TMS). After homogenization, 600 µL were transferred to 5 mm NMR tubes.

### 2.5. NMR Spectroscopy

NMR spectra were acquired on a Bruker AVANCE III spectrometer operating at 500.13 MHz for ^1^H. Unidimensional (1D) proton spectra were acquired at 298 K using the “noesypr1d” and “zg” pulse sequences (Bruker library, Rheinstetten, Germany), for aqueous and lipophilic extracts, respectively. Acquisition parameters may be found in reference [29]. Spectra were phased and baseline corrected manually, and chemical shifts were internally calibrated to TSP or TMS for aqueous and lipophilic extracts, respectively. To aid assignment, homonuclear (^1^H/^1^H) and heteronuclear (^1^H/^13^C) 2D NMR spectra [29] were acquired for selected samples. Peak assignment was also carried out based on existing the literature and databases (human metabolome database (HMDB) [61], Bruker BIOREFCODE (Bruker Biospin, Rheinstetten, Germany), and Chenomx NMR Suite (Chenomx Inc., Edmonton, AB, Canada)).

### 2.6. Data Processing and Statistical Analysis

Unidimensional ^1^H NMR spectra were converted into matrices (AMIX-viewer 3.9.14, Bruker Biospin, Rheinstetten, Germany), after excluding the water (δ 4.54–5.10) and methanol (singlet at δ 3.36) regions for aqueous extracts, and the chloroform region and corresponding satellite peaks (δ 7.00–7.50) for lipophilic extracts. All spectra were aligned (recursive segment-wise peak alignment (RSPA) (Matlab 8.3.0, The MathWorks Inc., Natick, MA, USA)) and normalized to total spectral area. Multivariate analysis was carried out by principal component analysis (PCA) and partial least-squares discriminant analysis (PLS-DA), as described previously [29]. Resonances exhibiting a clean profile, with no or minimal signal overlap, were integrated (Amix 3.9.14, Bruker BioSpin, Rheinstetten, Germany) and normalized to total spectral area. Metabolite levels varying significantly were identified by effect size (ES) [62] larger than ES error and *p* < 0.05, which were then qualitatively confirmed through direct inspection of the corresponding spectral regions. Statistical significance tests were carried out as described elsewhere [29]. The false discovery rate (FDR) correction, based on the Benjamini and Hochberg procedure [63], was applied to correct *p*-values for multiple comparisons.

## 3. Results

### 3.1. Impact of Pd_2_Spm on Mice Brain, Compared to cDDP: Polar Metabolome

**Figure 2:**
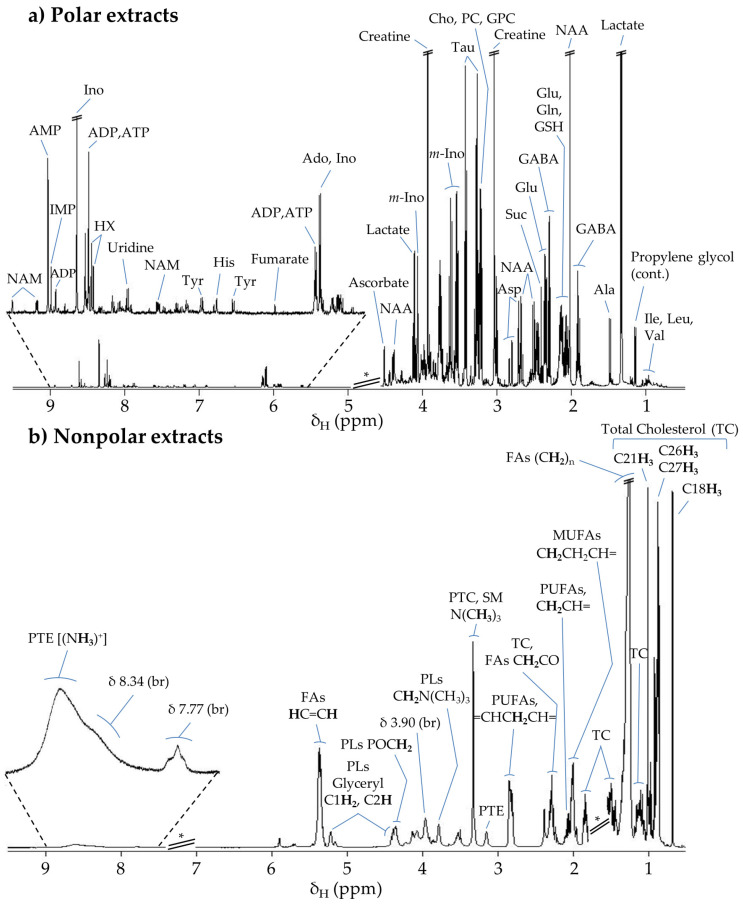
Average 500 MHz ^1^H NMR spectra of (**a**) polar and (**b**) nonpolar extracts of brain from healthy BALB/c mice at 1 h post-injection with phosphate-buffered saline solution (control group). * Cutoff spectral regions corresponding to water (δ 4.54–5.10) and residual CDCl_3_ and corresponding satellites (δ 7.00–7.50). Metabolite abbreviations: (**a**) 3-letter code used for amino acids; Ado, adenosine; ADP, adenosine diphosphate; AMP, adenosine monophosphate; ATP, adenosine triphosphate; Cho, choline; GABA, γ-aminobutyrate; GPC, glycerophosphocholine; GSH, glutathione (reduced); HX, hypoxanthine; IMP, inosine monophosphate; Ino, inosine; *m*-Ino, *myo*-Inositol; NAA, *N*-acetyl-aspartate; NAM, niacinamide; PC, phosphocholine; Suc, succinate; Tau, taurine. (**b**) FAs, fatty acids; MUFAs, monounsaturated fatty acids; PLs, phospholipids; PTC, phosphatidylcholine; PTE, phosphatidylethanolamine; PUFAs, polyunsaturated fatty acids; SM, sphingomyelin; TC, total cholesterol.

Figure 2a shows the average ^1^H NMR spectrum obtained for the polar extracts of the controls group, illustrating the predominance of lactate, *N*-acetyl-aspartate (NAA), and creatine, followed by a variety of amino acids, organic acids, and *m*-inositol resonating in the aliphatic region. In the expanded aromatic region, adenosine nucleotides and inosine seemed to predominate. Overall, forty-six metabolites have been identified in the polar extracts of mice brain (Table S1), in broad agreement with previous reports of other rodent models, where amino acids have mainly been identified, along with a few nucleotides and organic acids [59,64,65,66]. The present study adds information on the identification of nucleosides and purine derivatives (adenine and hypoxanthine, HX), as well as other compounds, e.g., acetone, dimethylamine (DMA), dimethyl sulfone (DMSO_2_), phosphoethanolamine (PE), and trimethylamine-*N*-oxide (TMAO) (^b^ in Table S1). These additions may reflect differences in the exact type of animal model, method of brain tissue sampling, or characteristics of the extraction protocol.

**Figure 3:**
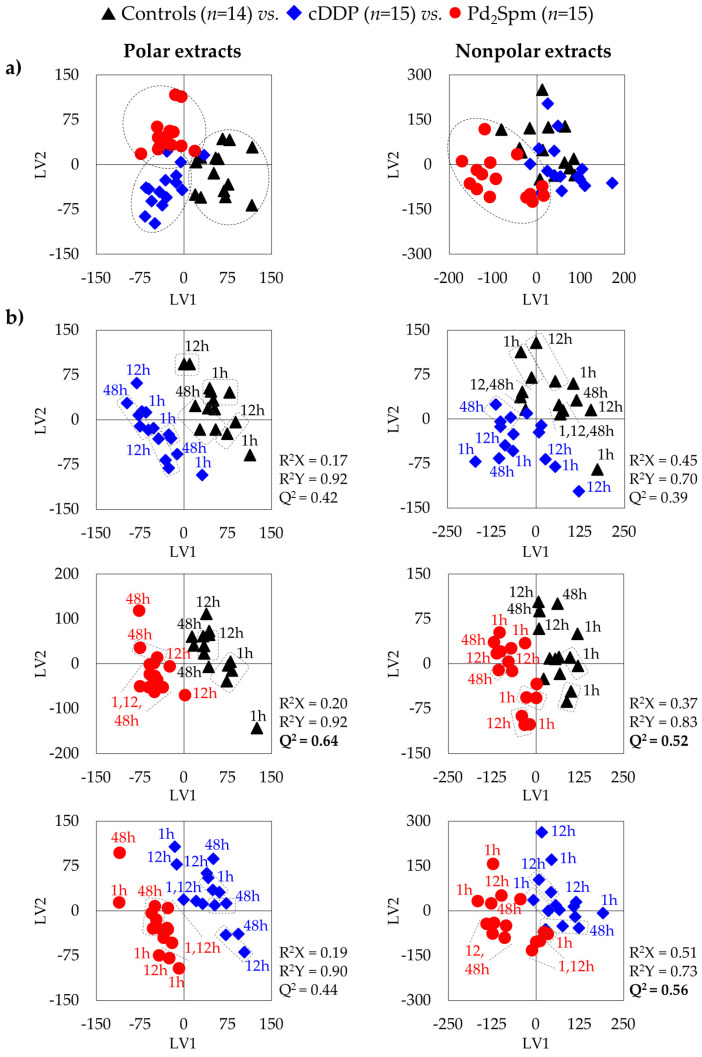
Score scatter plots of PLS-DA models for ^1^H NMR spectra of polar (**left**) and nonpolar (**right**) extracts of BALB/c mice brain, considering (**a**) all time-course samples for all three groups (controls, black triangles, *n* = 14; cDDP-treated, blue diamonds, *n* = 15; Pd_2_Spm-treated, red circles, *n* = 15), and (**b**) pairwise comparisons of cDDP-treated vs. controls, Pd_2_Spm-treated vs. controls, and Pd_2_Spm-treated vs. cDDP-treated. Post-injection time-points are specified for each sample. Validation parameters (R^2^ and Q^2^) are indicated for each pairwise model, with Q^2^ values > 0.5 highlighted in bold.

Comparison between the three animal groups (controls and cDDP- or Pd_2_Spm-exposed), aided by multivariate analysis, revealed a separation between all groups in the PLS-DA scores plot (Figure 3a, left). The corresponding PCA (not shown) showed large group overlap, reflecting significant sample dispersion, largely due to time-course variations. Indeed, most PCA (and PLS-DA) score plots showed clear group separation, when single post-injection times were considered (Figures S1 and S2, for cDDP and Pd_2_Spm exposure, respectively). Pairwise PLS-DA comparison of the full groups suggested a slightly more robust separation for Pd_2_Spm-treated samples, compared to controls (Q^2^ 0.64, Figure 3b, left), relatively to that characterizing the effect of cDDP (Q^2^ 0.42, Figure 3b, left). Some difference between the impacts of each drug is illustrated by separation noted when both drug-treated groups are compared directly (Q^2^ 0.44, Figure 3b, left).

**Figure 4:**
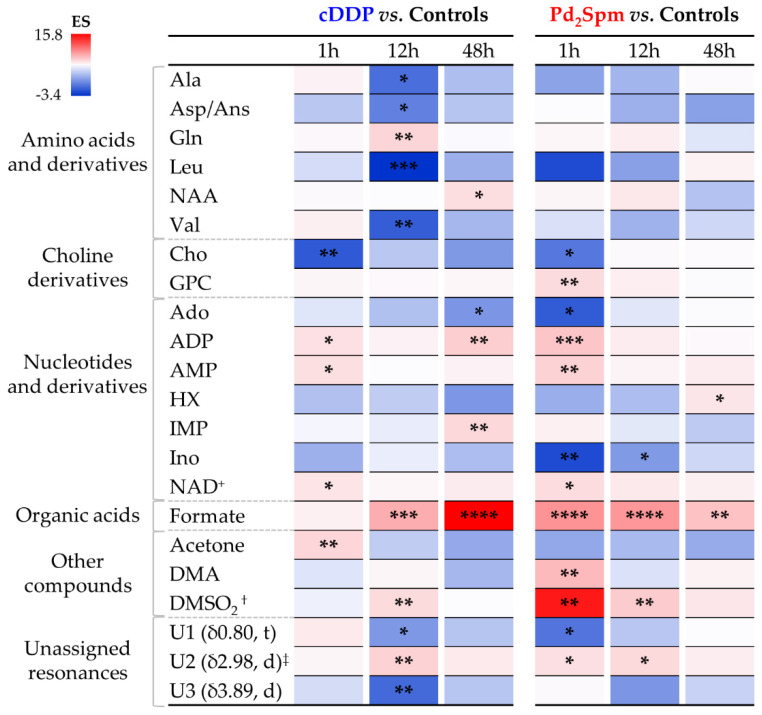
Heat map colored according to effect size of variations in the levels of polar metabolites, in the brain of healthy BALB/c mice, at 1, 12, and 48 h post-injection times either with cDDP or Pd_2_Spm, compared to controls. Abbreviations: NAD^+^, nicotinamide adenine dinucleotides; d, doublet; t, triplet; other abbreviations as defined in the caption of Figure 2. ^†^ Tentative assignment. ^‡^ Partial integration of resonance peak. * *p*-value < 5.0 × 10^−2^; ** *p*-value < 1.0 × 10^−2^; *** *p*-value < 1.0 × 10^−3^; **** *p*-value < 1.0 × 10^−4^ (asterisks correspond to comparison of each time point (for each drug) with controls).

**Figure 5:**
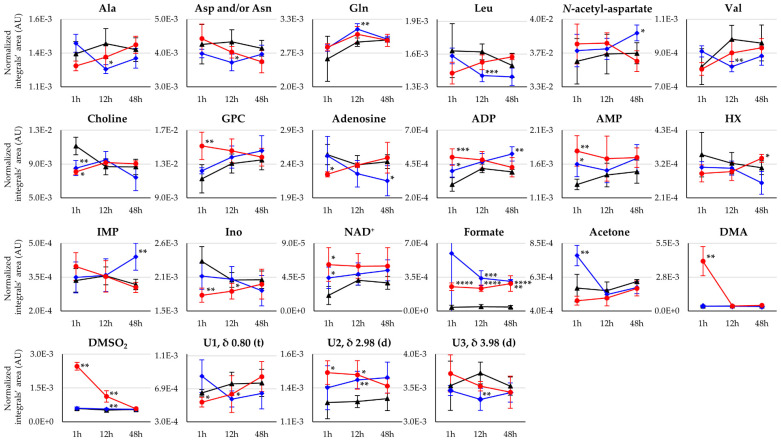
Selected time-course trajectory plots for polar metabolites related to cDDP- (blue line) or Pd_2_Spm-treated (red line) vs. controls (black line) mice brain tissue, comprising amino acids, choline derivatives, nucleotides/nucleosides and related compounds, organic acids, other compounds, and relevant unassigned resonances. Asterisks indicate the statistical significance of each drug compared only to controls at the indicated time point: * *p*-value < 5.0 × 10^−2^; ** *p*-value < 1.0 × 10^−2^; *** *p*-value < 1.0 × 10^−3^; **** *p*-value < 1.0 × 10^−4^.

The statistically relevant metabolite variations describing the impact of both metal complexes on the polar metabolome of mice brain, compared to controls, are listed in Table 1 and represented in a heat map format (Figure 4), where qualitative variation tendencies are also represented. The metabolite variations resulting from the direct comparison of the two drugs are listed in Table S2, top. Notably, Pd_2_Spm induced no significant changes in amino acids, compared to controls, whereas cDDP resulted in a marked general decrease at 12 h (affecting alanine, aspartate and/or asparagine, leucine, and valine), with the exceptions of increased glutamine and NAA levels (similar qualitative tendencies were also seen for Pd_2_Spm, but without statistical relevance). Inspection of the metabolite trajectory plots (Figure 5) confirms this, and enables the direct comparison of the drugs, with lines tending to cross over at 12 h (except for glutamine) and amino acid levels differentiating the two drugs mostly at 1 and 48 h (Table S2). As with other changes, these differences between drugs have always been confirmed by visual inspection of the spectra, as illustrated at 48 h for samples exposed to either cDDP or Pd_2_Spm (Figure S3). In addition, choline levels were lowered by both drugs at 1 h, compared to controls, following similar characteristics over the whole 1–48 h period; however, a glycerophosphocholine (GPC) increase clearly differentiated Pd_2_Spm at 1 h, from cDDP (where GPC evolution matches that of controls) (Figure 4 and Figure 5). In relation to nucleotides and derivatives, ADP, AMP, NAD^+^ levels were increased at 1 h by both drugs, but more significantly by Pd_2_Spm, along with early (1 h) stronger depletions in adenosine and inosine (Figure 4 and Figure 5). This stronger short-term impact of Pd_2_Spm on nitrogen bases and nucleotides may partially explain the slightly higher robustness of the Pd_2_Spm vs. the controls’ PLS-DA model; however, it is noted that most of these metabolites approximated control levels at 48 h (Figure 4). Statistically relevant Pd_2_Spm-specific nucleotide derivative variations comprised an early inosine decrease (1–12 h) and a later increase in HX (48 h); on the other hand, cDDP-treated brain tissue specifically exhibited a marked IMP increase at 48 h (Figure 4 and Figure 5). Both drugs induced elevated formate levels compared to controls (although effect size values (Table 1) were not, in this case, directly reflected in the trajectory plot (Figure 5), due to the significant standard deviation affecting the integral of the weak formate resonance); this is confirmed by no significant formate differences found in the direct comparison of the drug groups (Table S2).

The ketone body acetone specifically increased for cDDP at 1 h, with Pd_2_Spm inducing no relevant changes in this metabolite, compared to controls (Figure 5). Conversely, DMA and DMSO_2_ increase markedly early on (1–12 h) only for Pd_2_Spm. Notably, although both drugs affected the levels of DMSO_2_ (Figure 4), the effect is significantly stronger for Pd_2_Spm (Figure 5 and Table S2), most probably as a result of its required dissolution in 1% DMSO before injection, as previously discussed [29]. In general, the pattern of polar metabolite variations seems to show an earlier impact of Pd_2_Spm (including variations affecting some of the still unassigned resonances, Figure 4 and Figure 5), followed by almost complete recuperation at 48 h, with the exception of persisting increased levels of HX and formate (whereas altered levels of NAA, adenosine, ADP, IMP, and formate remain, at the same time point for cDDP). Indeed, the direct comparison of Pd_2_Spm- and cDDP-treated groups revealed a larger number of metabolite differences induced by the palladium complex at 1 h and at 48 h (Table S2, top), due to its corresponding earlier impact and faster recuperation of metabolite levels, respectively.

### 3.2. Impact of Pd_2_Spm on Mice Brain, Compared to cDDP: Nonpolar Metabolome

The average ^1^H NMR spectrum of control mice brain nonpolar extracts (Figure 2b) shows the predominance of cholesterol (mainly in its free form, with low levels of the esterified form), unsaturated fatty acids (FAs, including mono- and polyunsaturated FAs), and phospholipids (PL) (mostly phosphatidylcholine, PTC; phosphatidylethanolamine, PTE) (Table S1, bottom). This ^1^H NMR lipidic profile of brain adds to a previous ^1^H NMR report of brain nonpolar extracts [66] and reflects the fact that 40–75% of brain tissue dry weight is composed of lipids, 50–60% of which correspond to cholesterol and glycerophospholipids as structural components of cell membranes [67].

**Figure 6:**
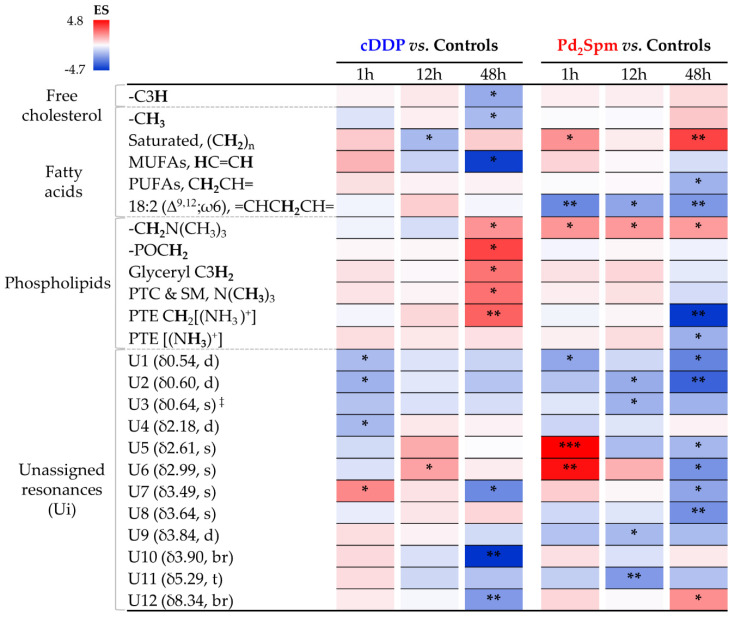
Heat map colored according to effect size of variations in the levels of nonpolar metabolites, in the brain of healthy BALB/c mice, at 1, 12, and 48 h post-injection times either with cDDP or Pd_2_Spm, compared to controls. Abbreviations: s, singlet; br, broad signal; other abbreviations as defined in Figure 2 and Figure 4. ^‡^ Partial integration of resonance peak. * *p*-value < 5.0 × 10^−2^; ** *p*-value < 1.0 × 10^−2^; *** *p*-value < 1.0 × 10^−3^ (asterisks correspond to comparison of each time point (for each drug) with controls).

**Figure 7:**
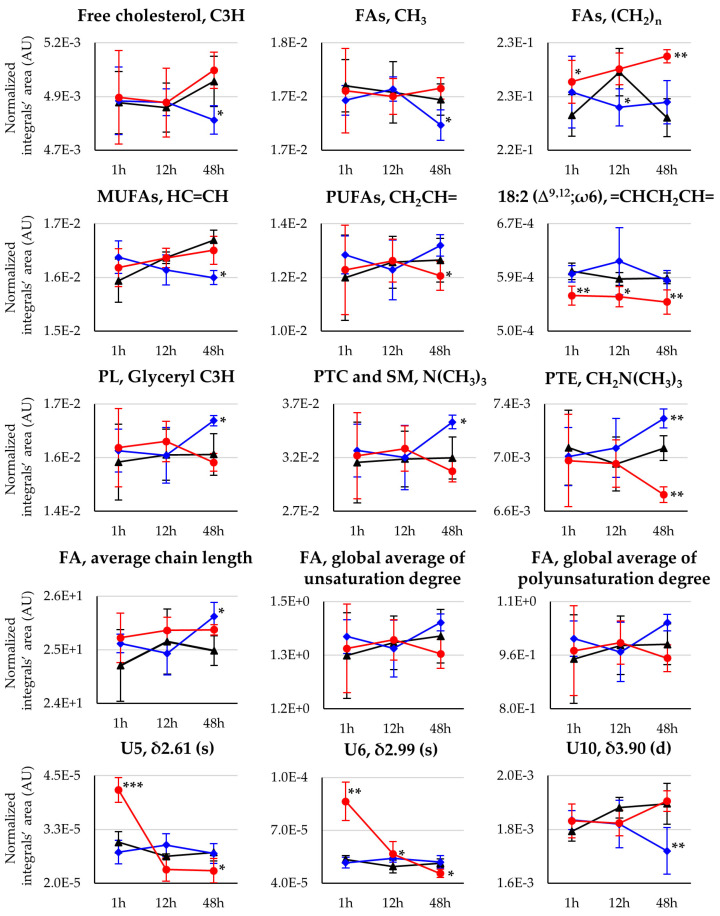
Selected time-course trajectory plots for nonpolar metabolites related to cDDP- (blue line) or Pd_2_Spm-treated (red line) vs. controls (black line) mice brain, comprising cholesterol, fatty acids (FAs), phospholipids (PLs), fatty acids average chain length, unsaturation and polyunsaturation degrees, and relevant unassigned resonances. Average FA chain length is expressed in terms of the (CH_2_)_n_/CH_3_ ratio, and average unsaturation and polyunsaturation degrees are expressed by the HC=CH/CH_3_ and =CHCH_2_CH=/CH_3_ ratios, respectively. Asterisks indicate the statistical significance of each drug compared only to controls at the indicated time point: * *p*-value < 5.0 × 10^−2^; ** *p*-value < 1.0 × 10^−2^; *** *p*-value < 1.0 × 10^−3^.

Multivariate analysis for comparison of the spectra of nonpolar extracts from the brain of controls, Pd_2_Spm- and cDDP-treated mice (Figure 3, right) showed similar results as observed for polar extracts (Figure 3, left), with PCA exhibiting differences between cDDP and Pd_2_Spm and controls only when analysis is restricted to a single time point (Figures S4 and S5, respectively). Pairwise PLS-DA score plots (Figure 3, right) again showed a slightly stronger effect (Q^2^ > 0.5) for Pd_2_Spm on the lipidic metabolome, compared to that of cDDP (Q^2^ 0.39), and a robust group separation (Q^2^ > 0.5) was noted between the groups treated with different drugs (Figure 3b, bottom). Signal integration and univariate analysis (Table 2 and Figure 6) showed that, firstly, the palladium complex does not lower free cholesterol levels, as it is clear for cDDP at 48 h, including from the time-course plot (Figure 7) and the direct comparison of drugs (Table S2, bottom). Secondly, global FA characteristics reflect distinct variations for CH_3_ and (CH_2_)_n_ resonances for the different drugs (Figure 6 and Figure 7 and Table S2), resulting, however, in similar average chain lengths at 48 h, which reflects a similar extent of longer FA biosynthesis compared to controls (see FA average chain length time-course plot in Figure 7). Regarding unsaturated FAs, Pd_2_Spm does not induce a decrease in MUFAs, which seems to result from cDDP treatment (Figure 6 and Figure 7), but rather it seems to reduce PUFAs preferentially (specifically including linoleic acid 18:2 Δ^9,12^). Hence, Pd_2_Spm treatment results in lower average unsaturation/polyunsaturation degrees, compared to both control and cDDP-treated tissues. In summary, although FAs were longer at all times in Pd_2_Spm-treated brain, relatively to controls (Figure 7), at 48 h the palladium complex induced FAs with similar average chain length and lower unsaturation degree compared to cDDP.

Regarding phospholipids (PLs), Pd_2_Spm triggered a very distinct profile from that characterizing cDDP treatment, namely comprising the absence of generalized increases at 48 h (as seen for cDDP, Figure 6) and a simultaneous marked decrease of PTE (Figure 7). In addition, a general increase in the global levels of choline containing PLs was observed at all times (Figure 6). Direct comparison of the two drugs (Table S2, bottom) shows that, in Pd_2_Spm-exposed brain tissue, PTC and SM levels were decreased and increased, respectively, at 48 h (notwithstanding the possible contribution of other unspecified choline PLs). As lipid resonances assignment is notably difficult in NMR, due to extensive overlap of lipid spin systems, a number of statistically relevant lipid resonances were identified as distinguishers of the drugs but still left unassigned (Table 2 and Table S2); however, their time-course pattern showed clear distinct patterns for each metal complex, compared to controls (Figure 6 and Figure 7), and between drugs directly (Table S2, bottom), thus contributing importantly to the distinct lipid signatures of brain metabolic response.

In terms of overall dynamics, both drugs seemed to induce strong changes in the lipidic metabolome throughout the whole 48 h period (Figure 6), with no clear dynamic distinctions between the two complexes. In addition, contrary to the polar metabolome, the changes observed in nonpolar compounds showed no signs of overall significant recovery at 48 h for either compound.

## 4. Discussion

To the best of our knowledge, this work adds novel and detailed information on the response of polar and nonpolar brain metabolomes to anticancer drugs in healthy mice. This information should be useful in assessing toxicity side effects of these agents on the healthy organism, together with a similar report on the liver, kidney, and breast tissue (the latter with relevance for breast cancer studies) of the same animals [29].

Besides the distinct detailed metabolite signatures of brain response to cDDP and Pd_2_Spm, the dynamics of such responses is a strong indicator of their distinct impacts on the brain. Notably, Pd_2_Spm exhibits a stronger early impact on the polar metabolome, which evolves to an almost complete recovery of control metabolite levels after 48 h, compared to cDDP, which exhibits a slower response, without recuperation, within the same time frame. On the other hand, nonpolar compounds remain altered throughout the 48 h period, with no signs of recuperation, for both drugs. The recuperation tendency of Pd_2_Spm-exposed animals, noted in the polar extracts, may be a reflection of the fact that, although small amounts of the metal ion accumulate in the brain of healthy mice compared to other organs (<1 ng/g) [11], stored Pd levels tend to more noticeably decrease over the 48 h period, whereas Pt levels remain apparently constant during the same period. This suggests that Pd-drugs may be better tolerated than Pt-drugs, although the reasons for the differences above (e.g., either related to biological uptake and/or different lability of metal species within the brain), are still unclear.

### 4.1. Amino Acids Metabolism

In relation to amino acids, our results have shown that only cisplatin affects the levels of brain amino acids with statistical significance, although it is interesting to note that Pd_2_Spm induces similar weaker qualitative changes. This suggests that an identical amino acid response is taking place, with cDDP exhibiting a stronger effect. The increase in NAA may relate to the decrease in the resonance assigned to asparagine and/or aspartate (where the latter is the more probable assignment), this acetylated form of aspartate being known to play important roles as a neuronal osmolyte [68], as well as a precursor of FAs and sterols [69]. Indeed, the diminished levels of free cholesterol observed in brain tissue exposed to cDDP (48 h) are consistent with the need to replenish the levels of this important sterol in the brain. Interestingly, the palladium complex does not seem to lead to cholesterol depletion (on the contrary, levels remain close to those in controls), indicating no disruption of cholesterol metabolism in the brain and, therefore, no particular need for the activation of aspartate to NAA conversion. Depletion of the branched chain amino acids (BCAA) leucine and valine is, again, statistically important for cDDP, and not for Pd_2_Spm. These are known to easily cross the BBB [70], due to their hydrophobicity, and function as nitrogen donors through their anapleurotic role in the tricarboxylic acid (TCA) cycle, for instance impacting on the glutamate/glutamine cycle [70]. This would explain the increase in glutamine levels (among the general decrease in amino acids), which is relevant for cDDP and only hinted at for Pd_2_Spm. The glutamate/glutamine cycle mediates the excitatory role of glutamate, within astrocytes and neurons, and acts as a protective mechanism, particularly of neurons, of glutamate-related excitotoxicity [70]. Interestingly, glutamate is readily detected in this work, but its levels remain constant through exposure time, which suggests the efficiency of the protective mechanisms in place. Glutamine also relates to amino acid exchange processes, to and from circulating blood [70], and (through glutamate) the synthesis of the neurotransmitter γ-aminobutyric acid (GABA) [70]. The latter compound is also detected in the spectra and observed to remain stable, again a possible indication that GABA regulation is efficiently achieved in brain cells. Such mechanisms seem to be rather subdued in Pd_2_Spm-exposed brain, possibly due to the noted relatively less extensive impact of this drug on amino acid metabolism, compared to cDDP. A recent account of the metabolic profile changes in the liver of the same animals [29] showed that Pd_2_Spm leads to a stronger early (1 h) depletion in several amino acids (including BCAAs), which suggests that amino acids may be mobilized from liver into circulation, to reach the brain and other organs, more effectively in the presence of Pd_2_Spm, so that (compared to cDDP) there is a lesser or no need for further amino acid depletion locally (in the brain), to mediate the processes described above.

### 4.2. Nucleotides’ Metabolism

In terms of nucleotides’ metabolism, compared to cDDP, the Pd_2_Spm complex induces earlier and more marked increases in ADP, AMP, NAD^+^, depletions in adenosine, along with apparent Pd_2_Spm-specific effects on inosine (1–12 h, decrease) and HX (increase). Except for the latter, all these disturbances recover to control levels from 12 h onwards. These results suggest that ADP and AMP pools’ enrichment may be occurring effectively upon 1 h of exposure to Pd_2_Spm, at the expense of adenosine (decreased at 1 h and at 48 h for Pd_2_Spm and cDDP, respectively) and ATP (observed not to vary) [71]. These pools are needed to feed the synthesis of nucleic acids, probably to compensate for DNA damage exerted by the drugs. The dynamics of this possible protective mechanism are distinctly different for the two drugs, with Pd_2_Spm acting quicker (1 h) and leading more rapidly to control levels of purine derivatives. In addition, HX may be playing an important antioxidant role in the brain, its levels remaining low (probably indicative of high oxidative stress), except for Pd_2_Spm-exposed tissue after 48 h. HX may be obtained from IMP [72] and we suggest that the higher levels of IMP for cDDP at 48 h indicate a stronger need for higher HX levels for suitable protection, whereas the decreasing tendency for HX in Pd_2_Spm is reversed from 12 to 48 h, with no need for more elevated IMP levels. These results, thus, suggest that Pd_2_Spm exposure seems to allow for a more effective antioxidant protection through HX, compared to cDDP. Interestingly, NAD^+^ levels vary similarly for both drugs, reflecting a similar NAD^+^/NADH-mediated regulation of general redox status [73]. It is, again, interesting to relate the above results to those obtained for nucleotides and derivatives in liver [29]. In animals exposed to cDDP, such metabolites were generally markedly increased after 48 h, compared to Pd_2_Spm, which suggests that higher amounts of nucleosides and nucleotides may be passed from liver into circulation to reach the brain, maybe to replenish purine levels in the cDDP-exposed brain.

### 4.3. Choline Compounds and Lipid Metabolism

Choline depletion in the mice brain exposed to each of the drugs may be indicative of gut microflora choline/betaine metabolism deviations and, perhaps more probably, related to cell membrane metabolism. In Pd_2_Spm-exposed brain tissue, this latter effect would be consistent with the specific GPC increase at 1 h (also increased in liver [29]) for Pd_2_Spm. This suggests an early disturbance in cell membrane biosynthesis, which is however rapidly returned to control levels [74].

Cholesterol metabolism differs significantly for the two drugs, with Pd_2_Spm not evidencing cholesterol depletion, as observed for cDDP. Cholesterol is a major component of cell membranes and its depletion with cDDP may reflect either membrane disruption or remodeling, and/or other deviant bioactive mechanisms mediated by this sterol [75]. If demonstrated to be related to the former effect in future studies, then it may be taken as an indicator of the extension of cell damage. The strong depletion induced by cDDP (but not by Pd_2_Spm) may trigger the use of NAA to replenish cholesterol levels, such not being as necessary with Pd_2_Spm. Cholesterol levels in the liver have been seen to increase as the result of exposure to either drug [29], thus suggesting a liver-mediated effort to provide the brain with replenished levels of cholesterol, possibly also along with raised formate levels (for both drugs), since cholesterol synthesis (in both liver and brain) also gives rise to formate [76]. It is possible that formate enters purine synthesis in the brain, [76], thus contributing to DNA repair mechanisms.

Other aspects of lipid metabolism in the brain include FA and PL metabolism, with both drugs inducing longer chain-length FAs compared to control tissue, however with lower average unsaturation/polyunsatuaration degrees for Pd_2_Spm-exposed brain tissue. This reflects the fact that Pd_2_Spm induces a preferential decrease in PUFAs, whereas cDDP triggers a decrease in MUFAs. This observation clearly demonstrates a distinct lipidic response to each drug, the origins of which call for further, more targeted, lipidomic and enzymatic studies. It is possible, however, that such observations may be correlated with the extent of oxidative stress (and its effect on the double bonds of unsaturated FAs) and the efficacy of protective mechanisms, suggested above to be more effective in the Pd_2_Spm-exposed brain. Furthermore, as FA composition may play important roles in lipid storage and determining membrane fluidity properties, the equilibrium status of saturated/unsaturated FAs may be of great importance. Interestingly, PL metabolism was shown to respond differently to the two drugs, with Pd_2_Spm inducing hardly any relevant PL changes (except for a strong decrease in PTE), whereas a generalized increase in several PL species characterized brain tissue when exposed to cDDP. This suggests that Pd_2_Spm seems to disturb membrane lipid metabolism less than cDDP, probably through a mechanism that tailors FA distribution to the required membrane fluidity characteristics. Another possible outcome of more saturated FAs as a response of the brain to Pd_2_Spm may be a higher ability for lipid energetic storage.

In addition, the ketone body acetone is elevated significantly only in the cDDP-exposed brain, which suggests more significant energy requirements at early exposure, compared to the Pd_2_Spm-exposed brain, as acetone may be obtained (together with other ketone bodies, although not detected here) and used to contribute to meeting energy requirements in the brain [77]. As both drugs lead to strong acetone depletion in the liver after 12 h (notably, stronger for cDDP than for Pd_2_Spm), it may be advanced that Pd_2_Spm-exposed brain tissue seems to use circulating or acetoacetate-derived acetone more rapidly (acetone levels already weakly depleted after 1 h) than cDDP-exposed tissue. Finally, a Pd_2_Spm-specific effect on DMA and DMSO_2_ levels has been noted before in the kidneys and liver of the same animals [29], consistently with the results noted here in the brain. Indeed, strong DMA and DMSO_2_ increases have been suggested as early markers of kidney response to the Pd_2_Spm complex [29], possibly related to choline conversion to betaine in the gut microflora, leading to DMA synthesis [78], and originating dimethylsulfide (DMS) and dimethylsulfoxide (DMSO), subsequently oxidized to DMSO_2_ [79]. Although it is probable that the extent of these effects will depend strongly on the required dissolution of Pd_2_Spm in 1% DMSO/water, it should be noted that DMSO_2_ levels, in particular, are also deviated by cDDP.

## 5. Conclusions

This work reports, for the first time to our knowledge, on the metabolic response of healthy mice brain to exposure to the potential new anticancer drug Pd_2_Spm, compared to cisplatin. In spite of the previously reported low accumulation of both metals in the brain, as compared to other organs, both drugs were found to impact significantly on brain metabolism, with Pd_2_Spm generally displaying a stronger early effect on purine nucleotides than cDDP, probably enhancing AMP and ADP pools for DNA repair and recovering control levels within 48 h. This seems to occur in tandem with an apparent more efficient synthesis of hypoxanthine from IMP, possibly for an improved oxidative stress protection. Furthermore, phospholipids seem to be less disturbed by Pd_2_Spm, which may indicate lesser membrane disruption and/or a more efficient mechanism of membrane protection, namely through mediation of cell membrane fluidity by the observed increased synthesis of more saturated fatty acids. Simultaneously, Pd_2_Spm induces no cholesterol depletion (as strongly observed for cDDP), which is again consistent with less cell membrane disruption, considering the important role of cholesterol as a structural membrane lipid in the brain. Furthermore, Pd_2_Spm induces hardly any changes in amino acids, contrary to cDDP, which seems to trigger NAA synthesis and BCAA use towards cholesterol replenishment and regulation of Glu/Gln cycle for glutamate excitotoxicity mediation. These mechanisms seem to be subdued in Pd_2_Spm-exposed brain, possibly benefiting from a more enhanced mobilization of amino acids from liver. Generally, these results suggest a more effective response of brain metabolism towards the possible detrimental effects of the potential anticancer drug Pd_2_Spm, in comparison with cDDP. Although the putative biochemical explanations advanced here require biochemical demonstration, they strongly suggest that the palladium drug may display a relatively more beneficial role than cDDP in relation to brain toxicity, which if demonstrated in breast cancer patients, may be encouraging for potential clinical applications of the Pd complex.

## Figures and Tables

**Table 1:** Significant metabolite variations (expressed in effect size, ES) in the polar metabolome of mice brain exposed to cDDP and Pd_2_Spm, compared to controls, at 1, 12, and 48 h post-injection times. Only variations with |ES| > ES error and *p*-value < 0.05 are shown. ^†^ Tentative assignment. ^‡^ Partial integration of resonance peak. ^a^ Metabolic variation statistically remaining significant after false discovery rate (FDR) correction. Metabolite abbreviations: 3-letter code used for amino acids; Ado, adenosine; ADP, adenosine diphosphate; AMP, adenosine monophosphate; Cho, choline; DMA, dimethylamine; DMSO_2_, dimethyl-sulfone; GPC, glycerophosphocholine; HX, hypoxanthine; IMP, inosine monophosphate; Ino, inosine; NAA, *N*-acetyl-aspartate; NAD^+^, nicotinamide adenine nucleotide; Ui, unassigned resonance. s, singlet; d, doublet; dd, doublet of doublets; t, triplet; q, quartet; m, multiplet.

			cDDP vs. Controls						Pd_2_Spm vs. Controls					
Metabolite Family/Assignment		δ_H_/ppm (Multiplicity)	1 h		12 h		48 h		1 h		12 h		48 h	
			ES ± Error	*p*-Value	ES ± Error	*p*-Value	ES ± Error	*p*-Value	ES ± Error	*p*-Value	ES ± Error	*p*-Value	ES ± Error	*P*-Value
Amino acids and derivatives	Ala	1.48 (d)	—	—	−2.3 ± 1.6	1.7 × 10^−2^	—	—	—	—	—	—	—	—
	Asp, Ans	2.80 (dd)	—	—	−2.0 ± 1.5	1.7 × 10^−2^	—	—	—	—	—	—	—	—
	Gln	2.45 (m)	—	—	2.7 ± 1.7	2.9 × 10^−3 a^	—	—	—	—	—	—	—	—
	Leu	0.96 (t)	—	—	−3.4 ± 1.9	8.5 × 10^−4 a^	—	—	—	—	—	—	—	—
	NAA	2.03 (s)	—	—	—	—	2.2 ± 1.7	2.1 × 10^−2^	—	—	—	—	—	—
	Val	1.05 (d)	—	—	−2.6 ± 1.7	8.8 × 10^−3^	—	—	—	—	—	—	—	—
Choline derivatives	Cho	3.20 (s)	−2.7 ± 1.7	2.9 × 10^−3 a^	—	—	—	—	−2.1 ± 1.6	1.3 × 10^−2^	—	—	—	—
	GPC	3.23 (s)	—	—	—	—	—	—	2.4 ± 1.6	5.8 × 10^−3 a^	—	—	—	—
Nucleotides and derivatives	Ado	4.29 (q)	—	—	—	—	−1.6 ± 1.5	4.6 × 10^−2^	−2.7 ± 1.7	1.1 × 10^−2^	—	—	—	—
	ADP	8.54 (s)	2.1 ± 1.5	1.2 × 10^−2^	—	—	3.2 ± 2.0	3.1 × 10^−3 a^	3.7 ± 2.0	4.8 × 10^−4 a^	—	—	—	—
	AMP	8.61 (s)	2.1 ± 1.6	1.8 × 10^−2^	—	—	—	—	2.9 ± 1.8	6.0 × 10^−3 a^	—	—	—	—
	HX	8.20 (s)	—	—	—	—	—	—	—	—	—	—	1.7 ± 1.5	3.2 × 10^−2^
	IMP	8.58 (s)	—	—	—	—	2.6 ± 1.8	7.2 × 10^−3^	—	—	—	—	—	—
	Ino	8.35 (s)	—	—	—	—	—	—	−3.0 ± 1.8	3.8 × 10^−3 a^	−1.5 ± 1.4	4.7 × 10^−2^	—	—
	NAD^+^	8.43 (s)	1.8 ± 1.5	2.3 × 10^−2^	—	—	—	—	2.3 ± 1.6	1.1 × 10^−2^	—	—	—	—
Organic acids	Formate	8.46 (s)	—	—	5.2 ± 2.6	5.8 × 10^−4 a^	15.7 ± 7.4	6.7 × 10^−8 a^	6.8 ± 3.2	3.3 × 10^−5 a^	6.5 ± 3.1	1.8 × 10^−5 a^	3.9 ± 2.2	2.0 × 10^−3^ ^a^
Other compounds	Acetone	2.24 (s)	2.7 ± 1.7	3.4 × 10^−3 a^	—	—	—	—	—	—	—	—	—	—
	DMA	2.73 (s)	—	—	—	—	—	—	4.4 ± 2.3	2.2 × 10^−3 a^	—	—	—	—
	DMSO_2_ ^†^	3.15 (s)	—	—	2.4 ± 1.6	7.9 × 10^−3^	—	—	14.3 ± 6.4	7.9 × 10^−3^	3.4 ± 1.9	5.8 × 10^−3^ ^a^	—	—
Unassigned resonances	U1	0.80 (t)	—	—	−1.5 ± 1.4	4.6 × 10^−2^	—	—	−2.2 ± 1.6	1.1 × 10^−2^	—	—	—	—
	U2	2.98 (d ^‡^)	—	—	2.9 ± 1.8	2.3 × 10^−3 a^	—	—	2.1 ± 1.6	1.2 × 10^−2^	2.4 ± 1.6	1.1 × 10^−2^	—	—
	U3	3.89 (d)	—	—	−2.4 ± 1.6	5.4 × 10^−3 a^	—	—	—	—	—	—	—	—

**Table 2:** Significant metabolite variations (expressed in effect size, ES) in the nonpolar metabolome of mice brain exposed to cDDP and Pd_2_Spm, compared to controls, at 1, 12, and 48 h post-injection times. Only variations with |ES| > ES error and *p*-value < 0.05 are shown. ^‡^ Partial integration of resonance peak. **^a^** Metabolic variation statistically significant even after false discovery rate (FDR) correction. Metabolite abbreviations: MUFAs, monounsaturated fatty acids; PTC, phosphatidylcholine; PTE, phosphatidylethanolamine; PUFAs, polyunsaturated fatty acids; SM, sphingomyelin; br, broad signal; other abbreviations as defined in Table 1.

			cDDP vs. Controls						Pd_2_Spm vs. Controls					
Metabolite Family/Assignment		δ_H_/ppm (Multiplicity)	1 h		12 h		48 h		1 h		12 h		48 h	
			ES ± Error	*p*-Value	ES ± Error	*p*-Value	ES ± Error	*p*-Value	ES ± Error	*p*-Value	ES ± Error	*p*-Value	ES ± Error	*p*-Value
Free cholesterol	C3**H**	3.53 (m)	—	—	—	—	−2.0 ± 1.6	3.2 × 10^−2^	—	—	—	—	—	—
Fatty acids	C**H_3_**	0.89 (br)	—	—	—	—	−1.6 ± 1.5	4.8 × 10^−2^	—	—	—	—	—	—
	Saturated, (C**H_2_**)_n_	1.25 (br)	—	—	−1.7 ± 1.4	3.2 × 10^−2^	—	—	1.8 ± 1.5	2.1 × 10^−2^	—	—	3.3 ± 2.0	2.2 × 10^−3^
	MUFAs, **H**C=C**H**	5.34 (m)	—	—	—	—	−4.5 ± 2.5	1.6 × 10^−2^	—	—	—	—	—	—
	PUFAs, C**H_2_**CH=	2.05 (m)	—	—	—	—	—	—	—	—	—	—	−1.8 ± 1.6	3.0 × 10^−2^
	18:2 (Δ^9,12^; ω6), =CHCH_2_CH=	2.77 (t)	—	—	—	—	—	—	−2.8 ± 1.7	2.4 × 10^−3^	−2.1 ± 1.6	1.3 × 10^−2^	−2.4 ± 1.7	8.8 × 10^−3^
Phospholipids	-C**H_2_**N(CH_3_)_3_	3.75 (br)	—	—	—	—	1.8 ± 1.6	3.5 × 10^−2^	1.8 ± 1.5	2.7 × 10^−2^	1.7 ± 1.5	4.1 × 10^−2^	1.7 ± 1.5	4.5 × 10^−2^
	-POC**H_2_**	4.38 (br)	—	—	—	—	3.3 ± 2.0	1.8 × 10^−2^	—	—	—	—	—	—
	Glyceryl C3**H_2_**	3.94 (br)	—	—	—	—	2.4 ± 1.7	4.4 × 10^−2^	—	—	—	—	—	—
	PTC & SM, N(C**H_3_**)_3_	3.29–3.31	—	—	—	—	2.5 ± 1.7	3.7 × 10^−2^	—	—	—	—	—	—
	PTE C**H**_2_[(NH_3_)^+^]	3.15 (br)	—	—	—	—	2.7 ± 1.8	9.0 × 10^−3^	—	—	—	—	−4.6 ± 2.5	1.5 × 10^−3^
	PTE [(N**H_3_**)^+^]	8.60 (br)	—	—	—	—	—	—	—	—	—	—	−1.9 ± 1.6	2.6 × 10^−2^
Unassigned resonances	U1	0.54 (d)	−1.6 ± 1.4	3.7 × 10^−2^	—	—	—	—	−2.1 ± 1.5	1.3 × 10^−2^	—	—	−2.9 ± 1.9	1.4 × 10^−2^
	U2	0.60 (d)	−1.8 ± 1.5	3.2 × 10^−2^	—	—	—	—	—	—	−1.9 ± 1.5	1.6 × 10^−2^	−3.7 ± 2.1	8.9 × 10^−3^
	U3	0.64 (s ^‡^)	—	—	—	—	—	—	—	—	−1.8 ± 1.5	2.1 × 10^−2^	—	—
	U4	2.18 (d)	−1.7 ± 1.4	3.6 × 10^−2^	—	—	—	—	—	—	—	—	—	—
	U5	2.61 (s)	—	—	—	—	—	—	4.5 ± 2.3	1.1 × 10^−4 a^	—	—	−1.7 ± 1.5	3.2 × 10^−2^
	U6	2.99 (s)	—	—	1.6 ± 1.4	4.6 × 10^−2^	—	—	4.2 ± 2.2	2.0 × 10^−3^	—	—	−2.5 ± 1.8	3.2 × 10^−2^
	U7	3.49 (s)	2.0 ± 1.5	1.4 × 10^−2^	—	—	−2.7 ± 1.8	1.5 × 10^−2^	—	—	—	—	−2.1 ± 1.6	4.6 × 10^−2^
	U8	3.64 (s)	—	—	—	—	—	—	—	—	—	—	−2.6 ± 1.8	6.0 × 10^−3^
	U9	3.84 (d)	—	—	—	—	—	—	—	—	−1.7 ± 1.4	3.1 × 10^−2^	—	—
	U10	3.90 (br)	—	—	—	—	−4.8 ± 2.6	1.2 × 10^−3^	—	—	—	—	—	—
	U11	5.29 (t)	—	—	—	—	—	—	—	—	−2.4 ± 1.6	5.9 × 10^−3^	—	—
	U12	8.34 (br)	—	—	—	—	−2.4 ± 1.7	8.4 × 10^−3^	—	—	—	—	1.9 ± 1.6	2.4 × 10^−2^

## Data Availability

Data available in a publicly accessible repository that does not issue DOIs. This data can be found on The Metabolomics Workbench, https://www.metabolomicsworkbench.org/ (accessed on 19 December 2021), with datatrack id numbers 3030 and 3031 for data of aqueous and lipophilic brain extracts, respectively.

## Supplementary Materials

The following are available online at https://www.mdpi.com/article/10.3390/pharmaceutics14020259/s1, Figure S1: PCA and PLS-DA score scatter plots for the ^1^H NMR spectra of polar extracts from brain of healthy BALB/c mice after exposure to cDDP at (a) 1 h, (b) 12 h, and (c) 48 h post-injections times, compared to controls; Figure S2: PCA and PLS-DA score scatter plots for the ^1^H NMR spectra of polar extracts from brain of healthy BALB/c mice after exposure to Pd_2_Spm at (a) 1 h, (b) 12 h, and (c) 48 h post-injections times, compared to controls; Figure S3: Examples of ^1^H NMR spectral regions illustrating changes in (a) polar and (b) nonpolar metabolites between cDDP-exposed (blue trace) and Pd_2_Spm-exposed (red trace), after 48 h of exposure; Figure S4: PCA and PLS-DA score scatter plots for the ^1^H NMR spectra of nonpolar extracts from brain of healthy BALB/c mice after exposure to cDDP at (a) 1 h, (b) 12 h, and (c) 48 h post-injections times, compared to controls; Figure S5: PCA and PLS-DA score scatter plots for the ^1^H NMR spectra of nonpolar extracts from brain of healthy BALB/c mice after exposure to Pd_2_Spm at (a) 1 h, (b) 12 h, and (c) 48 h post-injections times, compared to controls, Table S1: List of metabolites and corresponding spin systems identified in the 500 MHz ^1^H NMR spectra (of polar and nonpolar extracts of brain from healthy BALB/c mice, at 1 h post-injection time with phosphate buffered saline; Table S2: Significant metabolite variations (expressed in effect size, ES) in the polar (top) and nonpolar (bottom) metabolomes of mice brain exposed to Pd_2_Spm, compared to cDDP, at 1, 12, and 48 h post-injection times.
