# Supplementary material for: Metabolic Impact of Anticancer Drugs Pd2Spermine and Cisplatin on the Brain of Healthy Mice
Source: Pharmaceutics. 2022 Jan 22;14(2):259. doi: 10.3390/pharmaceutics14020259 (PMC8880159; doi:10.3390/pharmaceutics14020259)
Supplement: Supplementary file 1 [file pharmaceutics-14-00259-s001.zip › pharmaceutics-1540460-supplementary.pdf]

pharmaceutics

Supplementary Materials

Metabolic Impact of Anticancer Drugs Pd_2Spermine and Cisplatin on the Brain of Healthy Mice

Tatiana J. Carneiro [1], Martin Vojtek [2], Salomé Gonçalves-Monteiro [2], João R. Neves [1], Ana L.M. Batista de Carvalho [3], Maria Paula M. Marques [3,4], Carmen Diniz [2] and Ana M. Gil [1,*]

[1] Department of Chemistry and CICECO-Aveiro Institute of Materials, University of Aveiro, 3810-193 Aveiro, Portugal; tatiana.joao@ua.pt (T.J.C.), joaodrneves@ua.pt (J.R.N.)
[2] LAQV/REQUIMTE, Laboratory of Pharmacology, Department of Drug Sciences, Faculty of Pharmacy, University of Porto, 4150-755 Porto, Portugal; matovoj@gmail.com (M.V.), salomemonteiro8180@gmail.com (S.G.-M.), cdiniz@ff.up.pt (C.D.)
[3] Molecular Physical-Chemistry R&D Unit, Department of Chemistry, University of Coimbra, 3004-535 Coimbra, Portugal; almbc@uc.pt (A.L.M.B.d.C.), pmc@ci.uc.pt (M.P.M.M.)
[4] Department of Life Sciences, Faculty of Science and Technology, University of Coimbra, 3000-456 Coimbra, Portugal
* Correspondence: agil@ua.pt; Tel.: +351-234370707

Content of Supplementary Materials:

Figure S1. PCA and PLS-DA scores scatter plots for the ^{1}H NMR spectra of polar extracts from brain of healthy BALB/c mice after exposure to cDDP at a) 1 h, b) 12 h, and c) 48 h post-injections times, compared to controls.

Figure S2. PCA and PLS-DA scores scatter plots for the ^{1}H NMR spectra of polar extracts from brain of healthy BALB/c mice after exposure to Pd_2Spm at a) 1 h, b) 12 h, and c) 48 h post-injections times, compared to controls.

Figure S3. Examples of ^{1}H NMR spectral regions illustrating differences in a) polar and b) nonpolar metabolites between cDDP-exposed (blue trace) and Pd_2Spm-exposed (red trace), after 48 h of exposure.

Figure S4. PCA and PLS-DA scores scatter plots for the ^{1}H NMR spectra of nonpolar extracts from brain of healthy BALB/c mice after exposure to cDDP at a) 1 h, b) 12 h, and c) 48 h post-injections times, compared to controls.

Figure S5. PCA and PLS-DA scores scatter plots for the ^{1}H NMR spectra of nonpolar extracts from brain of healthy BALB/c mice after exposure to Pd_2Spm at a) 1 h, b) 12 h, and c) 48 h post-injections times, compared to controls.

Table S1. List of metabolites and corresponding spin systems identified in the 500MHz ^{1}H NMR spectra of polar and nonpolar extracts of brain from healthy BALB/c mice, at 1 h post-injection time with phosphate buffer saline.

Table S2. Significant metabolite variations (expressed in effect size, ES) in the polar (top) and nonpolar (bottom) metabolomes of mice brain exposed to Pd_2Spm, compared to cDDP, at 1, 12 and 48 h post-injection times.

Figure S1

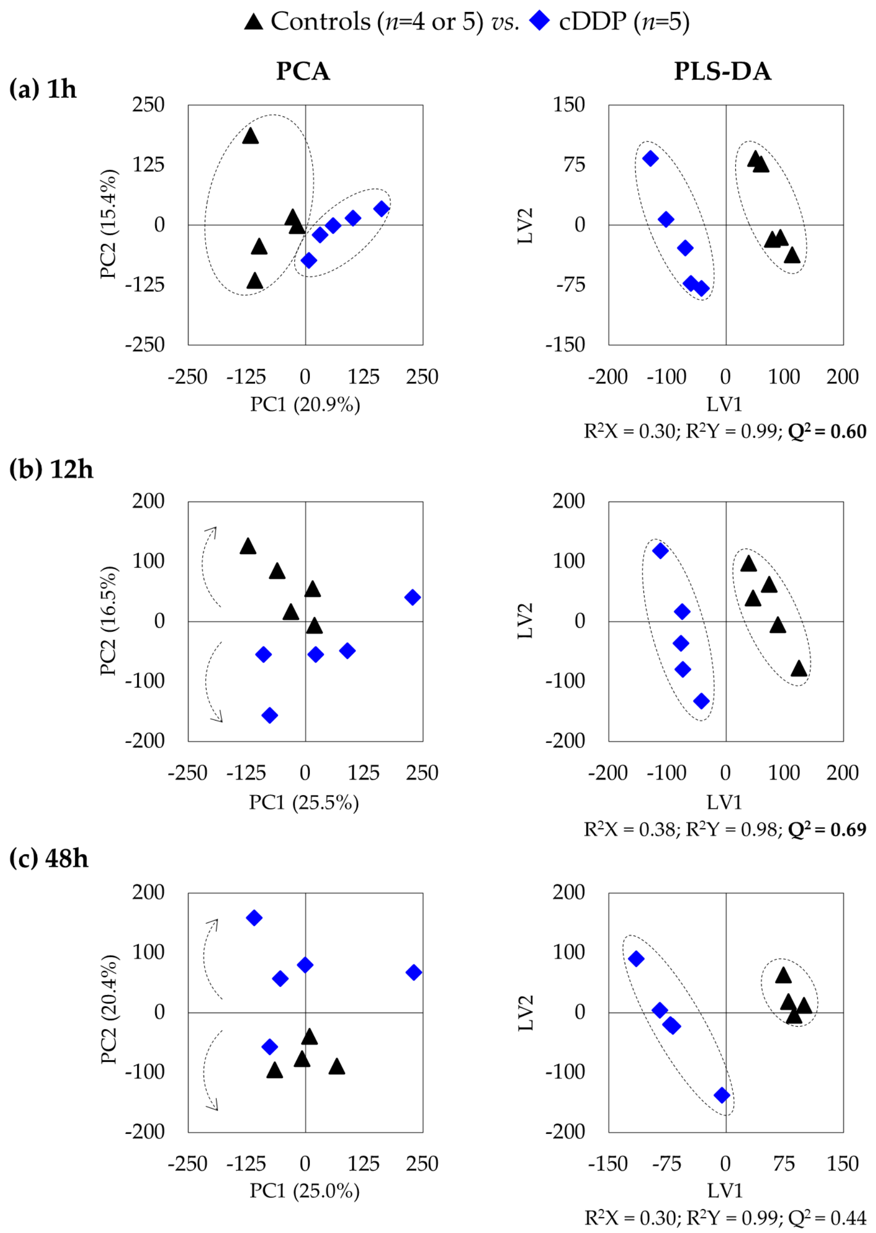

Figure S1. PCA and PLS-DA scores scatter plots for the ^{1}H NMR spectra of polar extracts from brain of healthy BALB/c mice after exposure to cDDP at a) 1 h, b) 12 h, and c) 48 h post-injections times, compared to controls. Validation parameters (R^2 and Q^2) are indicated for each PLS-DA model; Q^2 values > 0.5 are highlighted in bold.

Figure S2

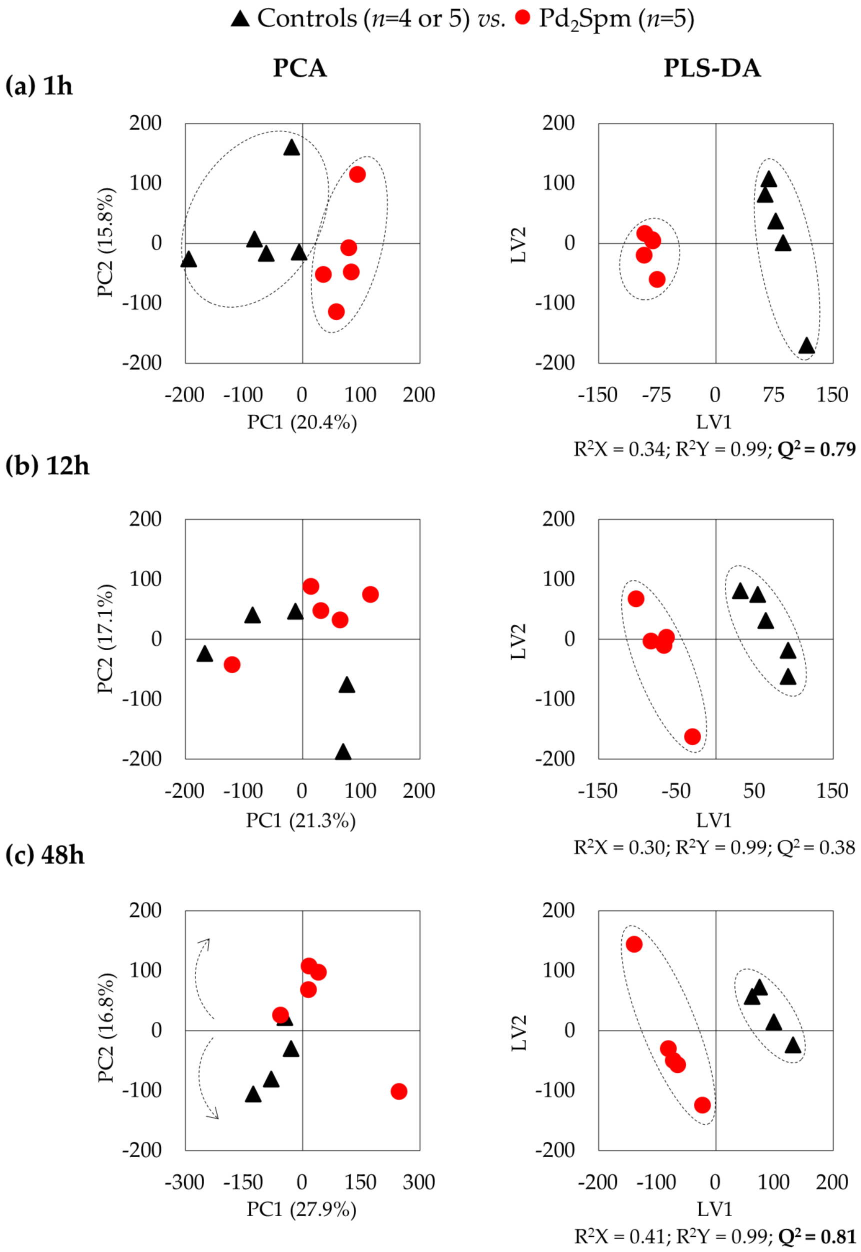

Figure S2. PCA and PLS-DA scores scatter plots for the ^1H NMR spectra of polar extracts from brain of healthy BALB/c mice after exposure to Pd_2Spm at a) 1 h, b) 12 h, and c) 48 h post-injections times, compared to controls. In PLS-DA models, Q^2 values > 0.5 (in bold) represent robust models.

Figure S3

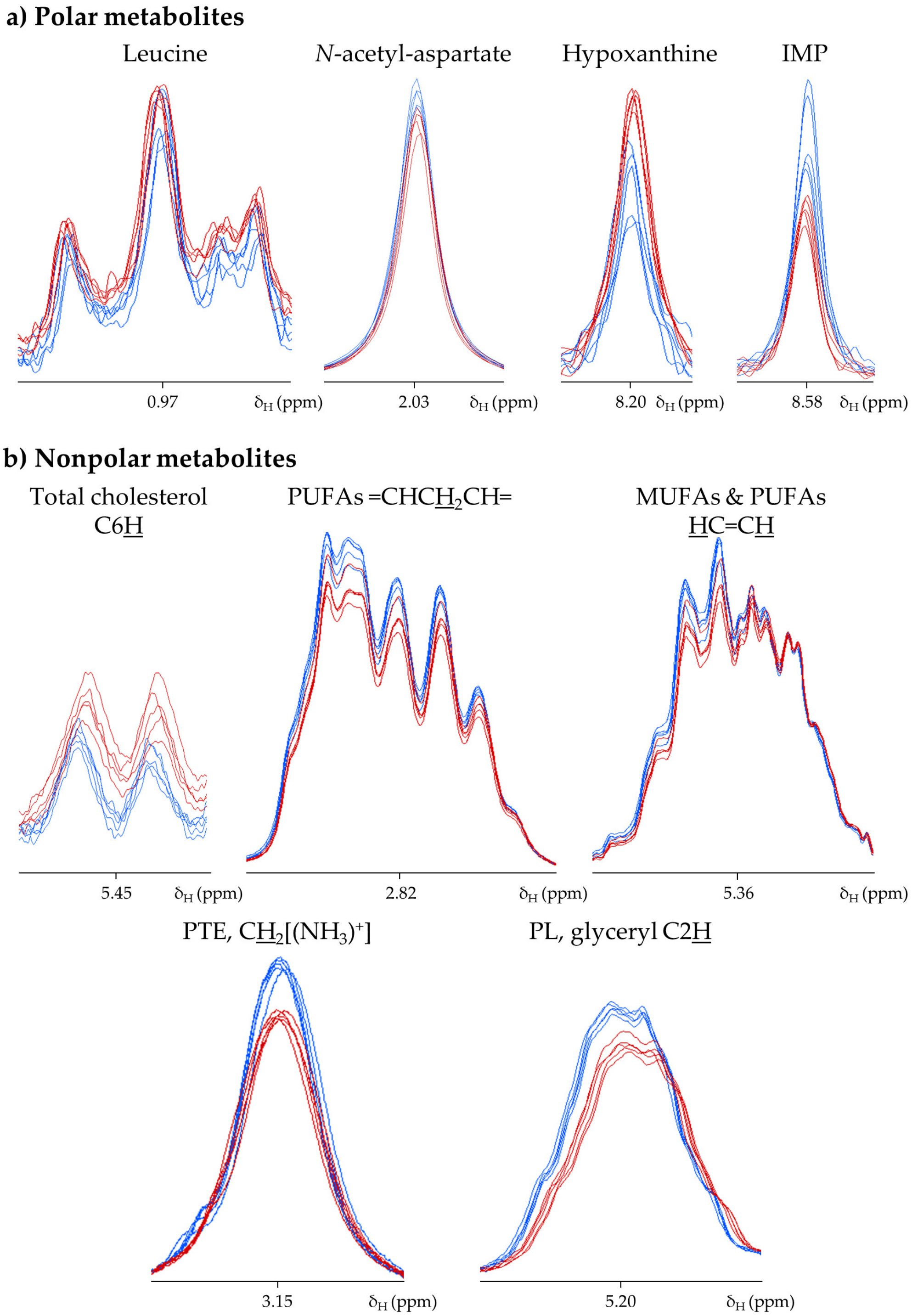

Figure S3. Examples of ^{1}H NMR spectral regions illustrating differences in a) polar and b) nonpolar metabolites between cDDP-exposed (blue trace) and Pd_2Spm-exposed (red trace), after 48 h of exposure.

Figure S4

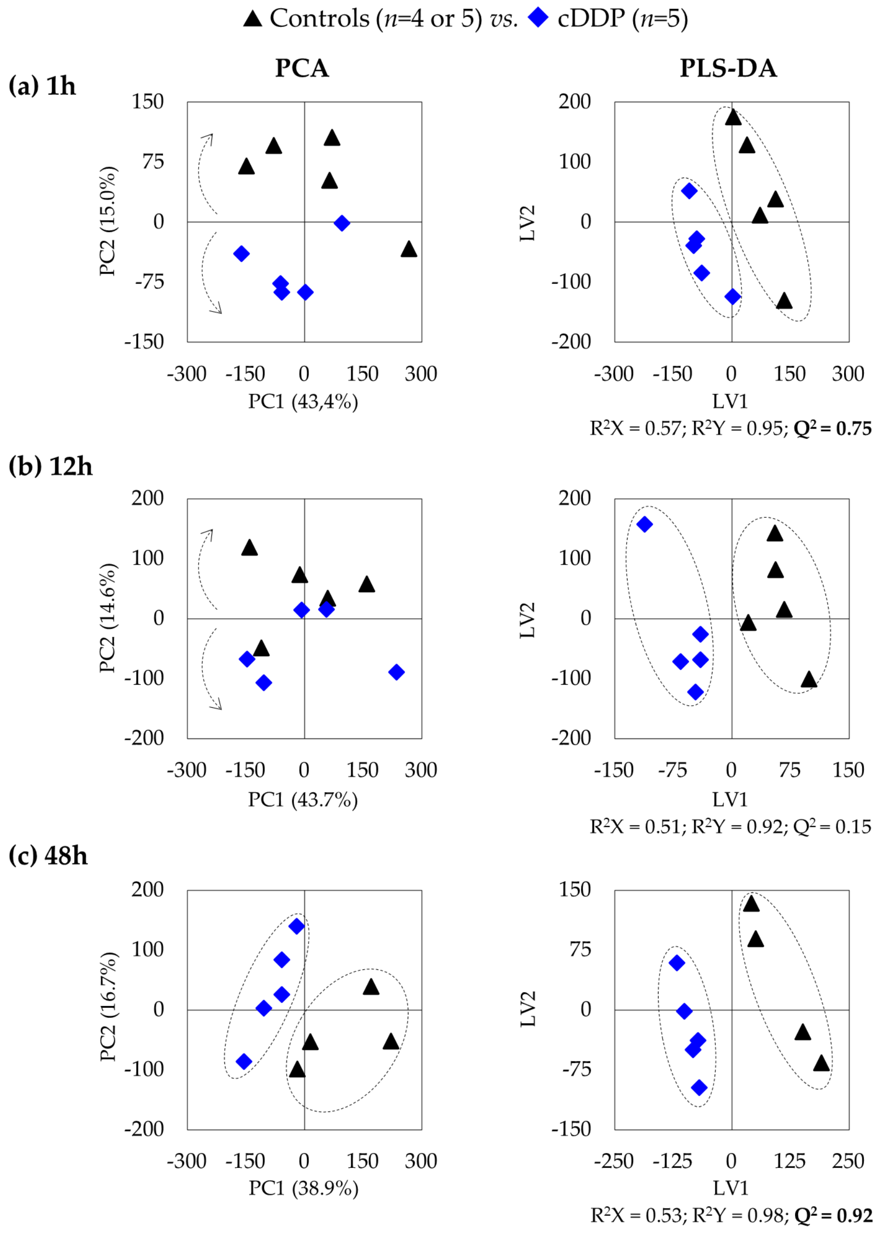

Figure S4. PCA and PLS-DA scores scatter plots for the ^{1}H NMR spectra of nonpolar extracts from brain of healthy BALB/c mice after exposure to cDDP at a) 1 h, b) 12 h, and c) 48 h post-injections times, compared to controls. In PLS-DA models, Q^2 values > 0.5 (in bold) represent robust models.

Figure S5

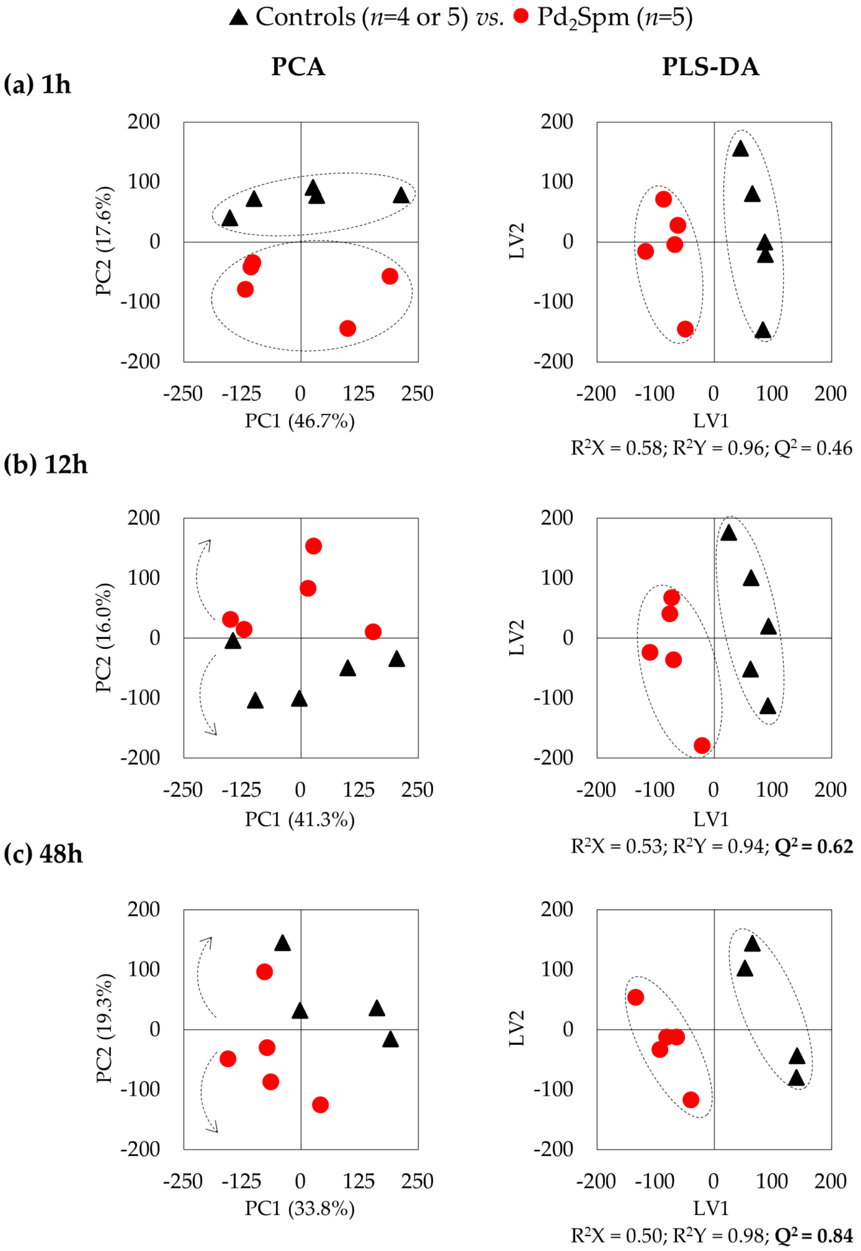

Figure S5. PCA and PLS-DA scores scatter plots for the ^{1}H NMR spectra of nonpolar extracts from brain of healthy BALB/c mice after exposure to Pd_2Spm at a) 1 h, b) 12 h, and c) 48 h post-injections times, compared to controls. In PLS-DA models, Q^2 values > 0.5 (in bold) represent robust models.

Table S1. List of metabolites and corresponding spin systems identified in the 500MHz ^{1}H NMR spectra of polar and nonpolar extracts of brain from healthy BALB/c mice, at 1 h post-injection time with phosphate buffer saline. [a] Predominant metabolites; [b] Metabolites identified, for the first time to our knowledge, by ^{1}H NMR in brain extracts of roedent models; * Possible contamination due to the extraction procedure; [†] Tentative assignment. Human Metabolome Database (HMDB ID) metabolite identidication codes are indicated whenever possible. Abbreviations: 3-HBA, 3-hydroxybutyrate; ADP, adenosine diphosphate; AMP, adenosine monophosphate; ATP, adenosine triphosphate; DMA, dimethylamine; DMSO$_2$, dimethyl sulfone; GABA, γ-aminobutyrate; GPC, glycerophosphocholine; GSH, glutathione (reduced); HX, hypoxanthine; IMP, inosine monophosphate; MUFAs, monounsaturated fatty acids; NAD$^+$, nicotinamide adenine dinucleotide; PC, phosphocholine; PE, phosphoethanolamine; PL, phospholipid; PTC, phosphatidylcholine; PTE, phosphatidylethanolamine; PUFAs, polyunsaturated fatty acids; SM, sphingomyelin; TMAO, Trimethylamine *N*-oxide; s, singlet; d, doublet; dd, double doublet; t, triplet; q, quartet; m, multiplet; br, broad signal.

	Metabolite	δ$_H$ ppm (multiplicity, assignment)	HMDB ID [61]
**Polar extracts**			
Amino acids and derivatives	Alanine	1.48 (d, βCH$_3$); 3.78 (q, αCH)	HMDB0000161
	Asparagine [†]	2.83 (m, βCH$_2$)	HMDB0000168
	Aspartate	2.68 (dd, βCH); 2.81 (dd, β′CH); 3.90 (dd, αCH)	HMDB0000191
	Creatine [a]	3.04 (s, N-CH$_3$); 3.93 (s, N-CH$_2$)	HMDB0000064
	GABA [a]	1.90 (m, βCH$_2$); 2.30 (t, γCH$_2$); 3.01 (t, αCH$_2$)	HMDB0000112
	Glutamate [a]	2.04 (m, βCH); 2.11 (m, β′CH); 2.36 (m, γCH$_2$); 3.76 (dd, αCH)	HMDB0000148
	Glutamine	2.14 (m, βCH$_2$); 2.45 (m, γCH$_2$); 3.77 (t, αCH)	HMDB0000641
	Glycine	3.55 (s, αCH$_2$)	HMDB0000123
	Histidine	7.06 (s, C4H ring); 8.00 (s, C2H ring)	HMDB0000177
	Isoleucine	0.94 (t, δCH3); 1.01 (d, β'CH3); 1.99 (m, βCH	HMDB0000177
	Leucine	0.96 (t, δCH$_3$/ δ′CH$_3$); 1.71 (m, βCH$_2$/ γCH)	HMDB0000687
	Lysine	1.45 (m, γCH$_2$); 1.73 (m, δCH$_2$); 1.92 (m, βCH$_2$)	HMDB0000182
	N-acetylaspartate [a]	2.02 (s, CH); 2.50, 2.70 (m, CH$_2$); 4.40 (m, CH$_3$)	HMDB0000812
	Phenylalanine	7.33 (d, C2H/ C6H ring); 7.38 (m, C4H ring); 7.42 (t, C3H/ C5H ring)	HMDB0000159
	Taurine [a]	3.27 (t, S-CH$_2$); 3.43 (t, N-CH$_2$)	HMDB0000251
	Tyrosine	6.90 (d, C3H/ C5H ring); 7.20 (d, C2H/ C6H ring)	HMDB0000158
	Valine	0.99 (d, γCH$_3$); 1.05 (d, γ'CH$_3$); 2.27 (m, βCH); 3.61 (d, αCH)	HMDB0000883
Choline deri- .	Choline [a]	3.21 (s, N(CH$_3$)$_3$)	HMDB0000097
	GPC	3.23 (s, N(CH$_3$)$_3$); 3.92 (m, αCH$_2$); 4.33 (m, PO$_3$-αCH$_2$)	HMDB0000086
	PC [a]	3.22 (s, N(CH$_3$)$_3$); 4.17 (m, PO$_3$-CH$_2$)	HMDB0001565
Nucleotides and derivatives	Adenine [b, †]	8.23 (s, C2H ring)	HMDB0000034
	Adenosine	4.29 (q, C4′H ribose); 4.44 (dd, C3′H ribose); 6.10 (d, C1′H ribose); 8.12 (s, C8H ring); 8.27 (s, C2H ring)	HMDB0000050
	ADP	6.15 (d, C1′H ribose); 8.27 (s, C2H ring); 8.54 (s, C8H ring)	HMDB0001341
	AMP	4.51 (dd, C2′H ribose); 6.14 (d, C1′H ribose); 8.27 (s, C2H ring); 8.61 (s, C8H ring)	HMDB0000045
	ATP	6.15 (d, C1′H ribose); 8.28 (s, C2H ring); 8.545 (s, C8H ring)	HMDB0000538
	Hypoxanthine [b]	8.18 (s, C2H); 8.20 (s, C8H)	HMDB0000157
	IMP	8.24 (s, C8H ring); 8.58 (s, C8H ring)	HMDB0000175
	Inosine	6.10 (d, C1′H); 8.24 (s, C8H ring); 8.35 (s, C2H ring)	HMDB0000195
	NAD$^+$ [†]	8.43 (s, A8)	HMDB0000902
	Uridine	5.90 (d, C5H ring); 5.92 (d, C1′H ring); 7.88 (d, C6H ring)	HMDB0000285
Or-gani	3-HBA	1.20 (d, CH$_3$); 2.31 (m, CH$_2$)	HMDB0000357
	Acetate	1.92 (s, CH$_3$)	HMDB0000042

	Ascorbate	4.52 (d, C4H ring)	HMDB0000044
	Formate	8.46 (s, CH)	HMDB0000142
	Fumarate	6.52 (s, CH)	HMDB0000134
	Lactate [a]	1.33 (d, CH_3); 4.10 (q, CH)	HMDB0000190
	Succinate	2.41 (s, CH_2)	HMDB0000254
Other compounds	Acetone [b]	2.24 (s, CH_3)	HMDB0001659
	DMA [b]	2.73 (s, $(CH_3)_2$)	HMDB0000087
	$DMSO_2$ [b, †]	3.15 (s, CH_3)	HMDB0004983
	Ethanol *	1.19 (t, CH_3); 3.65 (q, CH_2)	HMDB0000108
	m-Inositol [a]	3.28 (t, C5H); 3.54 (dd, C1H/ C3H) 3.62 (t, C4H/ C6H); 4.06 (t, C2H)	HMDB0000211
	Niacinamide	7.60 (m, C5H); 8.72 (m, C4H, C6H); 8.94 (m, C2H)	HMDB0001406
	PE [b, †]	3.99 (m, PO_3-CH_2)	HMDB0000224
	Propylene glycol *	1.15 (d, CH_3); 3.87 (m, CH)	HMDB0001881
	TMAO [b]	3.27 (s, CH_3)	HMDB0000925
Nonpolar extracts			
Cholesterol	Free [a]	0.68 (s, $C18H_3$); 0.86 (d, $C26H_3$); 0.87 (d, $C27H_3$); 0.91 (s, $C27H_3$); 0.95 (m, C9H); 0.99 (m, C14H); 1.01 (s, $C19H_3$); 1.07 (m, C1H); 1.10 (m, C17H); 1.12 (m, C23H/ C24H); 1.13 (m, C22H); 1.49 (m, C11H); 1.51 (m, C2H); 1.84 (m, C4H); 1.87 (t, C16H); 1.95 (t, C7H); 1.99 (t, C7H'); 2.26 (t, C4H'); 3.53 (m, C3H); 5.45 (d, C6H)	HMDB0000067
	Esterified	0.68 (s, $C18H_3$); 0.86 (d, $C26H_3$); 0.87 (d, $C27H_3$); 0.91 (s, $C27H_3$); 0.95 (m, C9H); 0.99 (m, C14H); 1.01 (s, $C19H_3$); 1.10 (m, C17H); 1.12 (m, C23H/ C24H); 1.13 (m, C22H); 1.15 (m, C1H); 1.84 (t, C4H); 1.87 (t, C15H'); 1.95 (t, C7H); 1.99 (t, C7H'); 2.31 (t, C4H'); 5.45 (d, C6H)	Undefined
Fatty acids (FA)	Saturated	0.89 (br, $\mathbf{CH_3}$); 1.25 (br, $(\mathbf{CH_2})_n$); 2.30 (m, $\mathbf{CH_2}$CO)	Undefined
	Monounsaturated	2.02 (ω7/ω9 $C\mathbf{H_2}CH_2CH$=); 2.30 (m, ω7/ω9 CH_2CO); 5.34 (m, **HC=CH**)	16:1 (Δ^9;ω7), HMDB0031053 / 18:1 (Δ^9;ω9), HMDB0000207
	Polyunsaturated [a]	0.93 (t, ω6 $\mathbf{CH_3}$); 0.98 (t, ω3 $\mathbf{CH_3}$); 1.30 (m, $(C\mathbf{H_2})_n$); 1.33 (m, ω3/ω6 =$CHCH_2(C\mathbf{H_2})_n$); 2.05 (m, $C\mathbf{H_2}CH$=); 2.30 (m, CH_2CO); 2.77 (t, 18:2 =$CHC\mathbf{H_2}CH$=); 2.81 (m, ω3/ω6 =$CHC\mathbf{H_2}CH$=); 2.82 (m, 22:6 =$CHC\mathbf{H_2}CH$=); 2.84 (m, 22:6 $\mathbf{CH_2}CO$); 5.37 (m, **HC=CH**)	18:2 ($\Delta^{9,12}$; ω6), HMDB0000673 22:6 ($\Delta^{4,7,10,13,16,19}$; ω3), HMDB0002183 and other undefined ω3/ω6 FAs
Phospholipids	PL resonances	3.75 (br, $\mathbf{CH_2}N(CH_3)_3$); 3.94 (br, Glyceryl $C3H_2$); 4.38 (br, $POC\mathbf{H_2}$); 4.40 (br, Glyceryl $C1H_2$); 5.20 (br, Glyceryl C2H)	Undefined
	PTC [a]	3.31 (s, $N(\mathbf{CH_3})_3$); 4.12 (m, Glyceryl, $C1\mathbf{H_2}$)	Undefined
	PTE [a]	3.15 (br, $\mathbf{CH_2}[(NH_3)^+]$); 8.60 (br, $[(N\mathbf{H_3})^+]$)	Undefined
	SM	3.29 (s, $N(\mathbf{CH_3})_3$)	Undefined

Table S2. Significant metabolite variations (expressed in effect size, ES) in the polar (top) and nonpolar (bottom) metabolomes of mice brain exposed to Pd_2Spm, compared to cDDP, at 1, 12 and 48 h post-injection times. Only variations with |ES| > ES Error and *p*-value < 0.05 are shown. † Tentative assignment. ‡ Partial integration of resonance peak. [a] Metabolic variation statistically significant after False Discovery Rate (FDR) correction; [b] Metabolite observed to vary only in the direct comparison of the two drugs (and not in comparison with controls). Abbreviations as defined in Tables 1 and S1.

			**Pd_2Spm *vs.* cDDP**								
	**Metabolite Family/ assignment**	**δ_H/ ppm (multiplicity)**	**1h**			**12h**			**48h**		
			ES	±Error	*p*-value	ES	±Error	*p*-value	ES	±Error	*p*-value
Amino acids and derivatives	Alanine	1.48 (d)	-3.2	1.9	2.4×10^{-3} [a]	—	—	—	1.5	1.4	4.9×10^{-2}
	Leucine	0.96 (t)	-1.8	1.5	2.4×10^{-2}	—	—	—	3.1	1.8	5.1×10^{-3} [a]
	N-acetylaspartate	2.03 (s)	—	—	—	—	—	—	-3.0	1.8	1.7×10^{-3} [a]
	Taurine [b]	3.43 (t)	—	—	—	—	—	—	-2.4	1.6	1.3×10^{-2}
	Valine	1.05 (d)	-3.0	1.8	1.7×10^{-3} [a]	—	—	—	—	—	—
Choline derivatives	Glycerophosphocholine	3.23 (s)	2.5	1.7	1.4×10^{-2}	—	—	—	—	—	—
	Phosphocholine [b]	3.21 (s)	—	—	—	—	—	—	-2.1	1.5	1.2×10^{-2}
Nucleotides and derivatives	Adenine †, [b]	8.23 (s)	—	—	—	—	—	—	-2.2	1.6	2.0×10^{-2}
	Adenosine	4.29 (q)	—	—	—	—	—	—	1.5	1.4	1.6×10^{-2}
	ADP	8.54 (s)	1.9	1.5	1.8×10^{-2}	—	—	—	-1.6	1.4	3.9×10^{-2}
	HX	8.20 (s)	—	—	—	—	—	—	2.9	1.8	6.2×10^{-3} [a]
	IMP	8.58 (s)	—	—	—	—	—	—	-3.0	1.8	4.8×10^{-3} [a]
	Inosine	8.35 (s)	-1.8	1.5	2.8×10^{-2}	—	—	—	—	—	—
Organic acids	Formate	8.46 (s)	—	—	—	-1.8	1.5	3.4×10^{-2}	—	—	—
Other compounds	Acetone	2.24 (s)	-5.7	2.8	1.7×10^{-3} [a]	—	—	—	—	—	—
	DMA	2.73 (s)	4.4	2.3	2.3×10^{-3} [a]	—	—	—	2.2	1.6	1.7×10^{-2}
	$DMSO_2$ †	3.15 (s)	14.5	6.5	7.9×10^{-3}	3.1	1.8	7.9×10^{-3}	—	—	—
Unassigned resonances	U1	0.80 (t)	-2.2	1.6	2.1×10^{-2}	—	—	—	—	—	—
	U4 [b]	2.06 (s‡)	—	—	—	—	—	—	2.4	1.6	3.2×10^{-2}
	U5 [b]	6.08 (s)	—	—	—	—	—	—	-1.8	1.5	4.5×10^{-2}
Cholesterol	Free, C3H	3.53 (m)	—	—	—	—	—	—	3.1	1.8	7.9×10^{-3} [a]
	Total, C6H	5.45 (d)	—	—	—	—	—	—	3.2	1.9	2.7×10^{-3} [a]
	Total, C7H & C7H′	1.95-1.97	—	—	—	—	—	—	1.9	1.5	2.5×10^{-2}
	Total, $C26H_3$	0.86 (d)	—	—	—	—	—	—	1.8	1.5	2.8×10^{-2}
	Total, $C27H_3$	0.87 (d)	—	—	—	—	—	—	4.3	2.3	1.6×10^{-4} [a]
Fatty acids	$\mathbf{CH_3}$	0.89 (br)	—	—	—	—	—	—	2.8	1.7	7.9×10^{-3} [a]
	Saturated, $(\mathbf{CH_2})_n$	1.25 (br)	2.8	1.7	7.9×10^{-3} [a]	—	—	—	2.3	1.6	7.2×10^{-3} [a]
	MUFAs (Δ^9; ω7/ω9), $\mathbf{CH_2}CH_2CH=$	2.02 (m)	—	—	—	—	—	—	1.9	1.5	2.1×10^{-2}
	MUFAs, **HC=CH**	5.34 (m)	—	—	—	—	—	—	2.5	1.6	7.9×10^{-3} [a]
	PUFAs (ω3), CH_3	0.98 (t)	—	—	—	—	—	—	-1.6	1.4	3.6×10^{-2}
	PUFAs, $(\mathbf{CH_2})_n$	1.30 (m)	—	—	—	—	—	—	2.3	1.6	1.1×10^{-2} [a]
	PUFAs (ω3/ω6), $=CHCH_2(\mathbf{CH_2})_n$	1.33 (m)	—	—	—	—	—	—	3.4	1.9	9.9×10^{-4} [a]
	PUFAs, $\mathbf{CH_2}CH=$	2.05 (m)	—	—	—	—	—	—	-1.9	1.5	1.6×10^{-2} [a]
	PUFAs (ω3/ω6), $=CH\mathbf{CH_2}CH=$	2.81 (m)	—	—	—	—	—	—	-2.4	1.6	6.5×10^{-3} [a]
	PUFAs, **HC=CH**	5.37 (m)	—	—	—	—	—	—	-2.7	1.7	3.0×10^{-3} [a]
	18:2 ($\Delta^{9,12}$; ω6), $=CH\mathbf{CH_2}CH=$	2.77 (t)	-2.5	1.7	4.29×10^{-3} [a]	-1.3	1.4	1.6×10^{-2} [a]	-2.0	1.5	1.5×10^{-2} [a]
	22:6 ($\Delta^{4,7,10,13,16,19}$; ω3), $\mathbf{CH_2}CO$	2.38 (m)	—	—	—	—	—	—	-3.8	2.1	3.3×10^{-4} [a]
	22:6 ($\Delta^{4,7,10,13,16,19}$;ω3), $=CH\mathbf{CH_2}CH=$	2.82 (m)	—	—	—	—	—	—	-3.1	1.8	1.4×10^{-3} [a]
Phospholipids	$\mathbf{CH_2}N(CH_3)_3$	3.75 (br)	2.3	1.6	8.9×10^{-3} [a]	1.7	1.4	3.4×10^{-2}	—	—	—
	$PO\mathbf{CH_2}$	4.38 (br)	—	—	—	—	—	—	-5.9	2.9	1.2×10^{-4} [a]
	Glyceryl $C3\mathbf{H_2}$	3.94 (br)	—	—	—	—	—	—	-5.9	2.9	5.8×10^{-5} [a]
	Glyceryl $C1\mathbf{H_2}$	4.40 (br)	—	—	—	—	—	—	-4.1	2.2	2.0×10^{-4} [a]
	Glyceryl C2**H**	5.20 (br)	—	—	—	—	—	—	-4.9	2.5	8.9×10^{-5} [a]
	PTC & SM, $N(\mathbf{CH_3})_3$	3.29 - 3.31	—	—	—	—	—	—	-5.6	2.7	5.7×10^{-5} [a]
	PTC, Glyceryl $C1\mathbf{H_2}$	4.12 (m)	—	—	—	—	—	—	-3.4	2.0	7.0×10^{-4} [a]

	PTE C**H**$_2$[(NH$_3$)$^+$]	3.15 (br)	—	—	—	—	—	—	-8.8 ± 4.1	9.9×10^{-7} [a]
	PTE [(N**H**$_3$)$^+$]	8.60 (br)	—	—	—	—	—	—	-2.3 ± 1.6	7.4×10^{-3} [a]
	SM	5.70 (m)	—	—	—	—	—	—	2.8 ± 1.7	4.0×10^{-3} [a]
Unassigned resonances	U2	0.60 (d)	—	—	—	—	—	—	-2.3 ± 1.6	1.1×10^{-2} [a]
	U5	2.61 (s)	5.2 ± 2.6		3.9×10^{-5} [a]	-2.2 ± 1.6		9.4×10^{-3} [a]	-1.6 ± 1.4	4.0×10^{-2}
	U6	2.99 (s)	4.4 ± 2.3		1.3×10^{-3} [a]	—	—	—	-2.1 ± 1.5	1.6×10^{-2} [a]
	U9	3.84 (d)	-1.8 ± 1.5		2.8×10^{-2}	—	—	—	— —	—
	U10	3.90 (br)	—	—	—	—	—	—	5.5 ± 2.7	3.4×10^{-5} [a]
	U11	5.29 (t)	—	—	—	-2.0 ± 1.5		1.6×10^{-2} [a]	— —	—
	U12	8.34 (br)	—	—	—	—	—	—	4.0 ± 2.2	2.2×10^{-4} [a]
	U13	0.89 (s)	—	—	—	—	—	—	1.8 ± 1.5	2.6×10^{-2}
	U14	2.20 (q)	—	—	—	—	—	—	2.5 ± 1.7	4.0×10^{-3} [a]
	U15	4.22 (br)	—	—	—	—	—	—	3.5 ± 2.0	1.4×10^{-3} [a]
	U16	7.77 (br)	—	—	—	—	—	—	2.7 ± 1.7	4.4×10^{-3} [a]
